# Towards an Asymmetric Organocatalytic α-Azidation of β-Ketoesters

**DOI:** 10.3390/molecules23051142

**Published:** 2018-05-11

**Authors:** Maximilian Tiffner, Lotte Stockhammer, Johannes Schörgenhumer, Katharina Röser, Mario Waser

**Affiliations:** Institute of Organic Chemistry, Johannes Kepler University Linz, Altenbergerstr. 69, 4040 Linz, Austria; maximilian.tiffner@gmail.com (M.T.); lotte.stockhammer@gmail.com (L.S.); j.schoergenhumer@gmail.com (J.S.); ka.roeser@aon.at (K.R.)

**Keywords:** azides, organocatalysis, hypervalent iodine reagents, ammonium iodides, cinchona alkaloids

## Abstract

Detailed investigations concerning the organocatalytic (asymmetric) α-azidation of prochiral β-ketoesters were carried out. It was shown that the racemic version of such a reaction can either be carried out under oxidative conditions using TMSN_3_ as the azide-source with quaternary ammonium iodides as the catalysts, or by using hypervalent iodine-based electrophilic azide-transfer reagents with different organocatalysts. In addition, the latter strategy could also be carried out with modest enantioselectivities when using simple cinchona alkaloid catalysts, albeit with relatively low yields.

## 1. Introduction

**Scheme 1:**
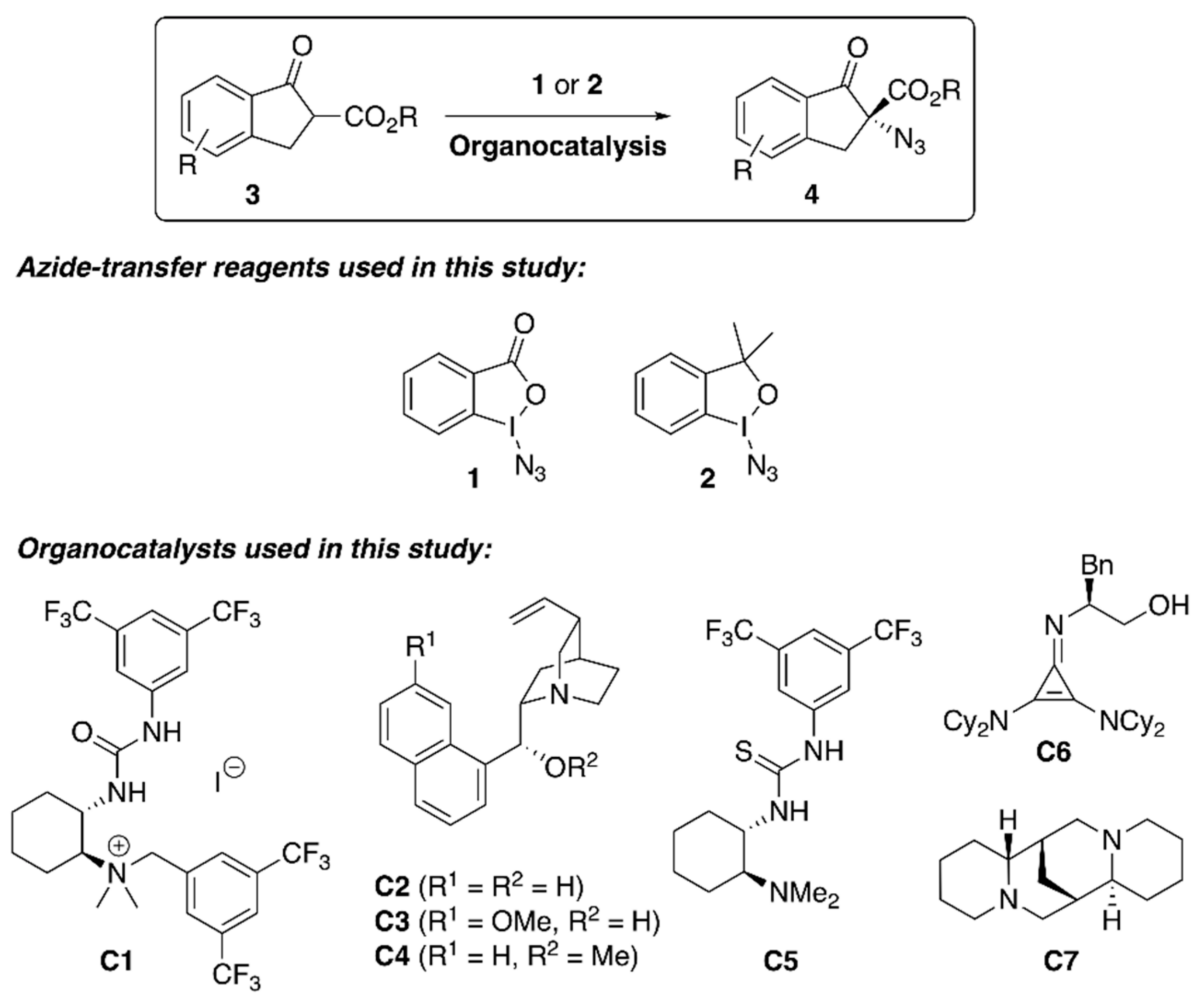
Targeted α-azidation of β-ketoesters **3** using the electrophilic azide-transfer reagents **1** and **2**.

Organic azides are an important and versatile class of molecules, and their syntheses and applications have attracted considerable interest [1,2,3,4,5,6,7,8,9,10]. Their potential lies in the fact that these compounds can serve as valuable building blocks for further transformations, e.g., the direct syntheses of heterocycles by means of a 1,3-dipolar cycloaddition with alkynes [3,4,5], or their straightforward reduction to free amines [6,7,8] (to mention only a few thoroughly investigated prominent applications of azides). Keeping this high potential in mind, it is no surprise that a variety of strategies to introduce azide functionalities in organic molecules have been reported [1,7,8,9,10]. The classical way of installing an azide group is based on the displacement of leaving groups, e.g., halides, by nucleophilic azide sources, like NaN_3_ or trimethylsilylazide (TMSN_3_) [11]. In addition, the recent years there has also been remarkable progress in using these nucleophilic azides under oxidative conditions, which allows for the azidation of usually-nucleophilic species, e.g., enolates, by using bench-stable N_3_^−^ reagents under operationally simple conditions [12,13,14,15]. An alternative strategy is based on the use of hypervalent iodine-based electrophilic azide-transfer reagents, e.g., compounds **1** and **2** (Scheme 1) [16,17,18,19,20]. The use of hypervalent iodine reagents for the transfer of different “uncommon” electrophiles has emerged as a powerful method over the last few years [16,17] and the use of reagents **1** and **2** to facilitate the α-azidation of prochiral nucleophiles represents an impressive application of such reagents. Pioneering reports by the groups of Gade [20] and Waser [19] have shown that α-azidations of enolate species with compounds **1** and **2** is possible, even in a highly asymmetric fashion under chiral iron catalysis [20]. It was recently shown that hypervalent iodine-based electrophile-transfer reagents can also be successfully used for a variety of asymmetric α-functionalization reactions of prochiral nucleophiles under non-covalent organocatalysis (e.g., ion pairing catalysis, organobase catalysis, etc.) [17,21,22,23,24]. Surprisingly, however, the asymmetric α-azidation of commonly-employed β-ketoesters **3** has, to the best of our knowledge, not been systematically investigated using chiral non-covalent organocatalysis so far. Based on our research focus on asymmetric α-heterofunctionalization reactions [25,26,27,28], as well as our interest in the use of hypervalent iodine reagents under organocatalytic conditions [24], we have, therefore, now carried out detailed investigations of the α-azidation of ketoesters **3** with the electrophilic azide-transfer reagents **1** and **2** using the chiral organocatalysts **C1**–**C7** shown in Scheme 1 [26,29,30,31,32].

**Scheme 2:**
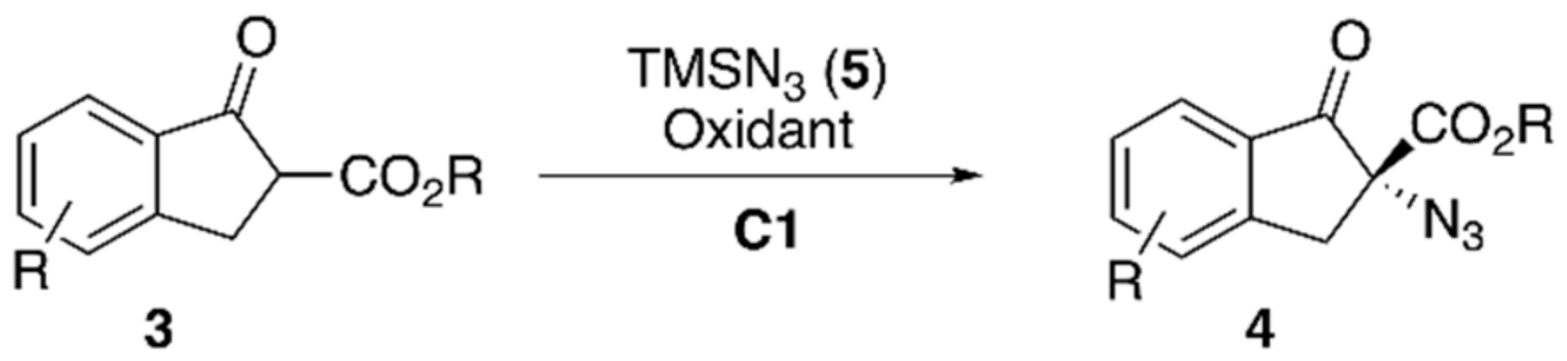
Targeted α-azidation of β-ketoesters **3** using TMSN_3_ under oxidative conditions using the chiral ammonium iodide catalyst **C1**.

Another strategy that attracted our interest is the use of simple nucleophilic azide reagents, like TMSN_3_ (**5**), in combination with oxidizing agents in the presence of achiral ammonium or guanidinium iodides to facilitate the α-azidation of carbonyl compounds [14,15]. Inspired by these studies and recent reports that showed that chiral ammonium iodides can be used for asymmetric oxidative transformations [33], we also wanted to test if an enantioselective α-azidation by using our bifunctional ammonium iodide catalyst **C1** in combination with TMSN_3_ and an oxidant may allow us to establish an enantioselective protocol for the synthesis of azides **4** (Scheme 2).

## 2. Results and Discussion

We started our investigations by carrying out the α-azidation of ketoester **3a** with the hypervalent iodine reagent **1** under a variety of conditions using different organocatalysts (Table 1 gives an overview of the most significant results of a rather broad screening). First attempts were carried out with our bifunctional ammonium salt catalyst **C1** [26,27,28] (entries 1–4). In all experiments where we used either an inorganic or an organic base we were able to isolate the product **4a** in good yields in relatively short reaction times but, unfortunately, with no asymmetric induction. When we carried out the reaction under base-free conditions instead (entry 2), the conversion dropped significantly but, again, no enantioselectivity could be obtained. Unfortunately, we were also not able to achieve any mentionable face-differentiation under ammonium salt catalysis by using other solvents, bases, or catalyst derivatives and, thus, switched our focus on the easily-accessible chiral amines **C2**–**C4** next (entries 5–16). The first experiment already showed that cinchonidine (**C2**) is able to control the azidation, albeit the yield was rather low herein (entry 5). By increasing the catalyst loading to 40 mol% (entry 6), the yield and the enantioselectivity could be increased but, unfortunately, the conversion was still limited to around 50% (with 44% isolated yield). Neither prolonging the reaction time nor carrying out the azidation at higher temperature (entry 7) allowed us to achieve a higher product yield. One possible explanation for this reduced turnover could be that the formed iodobenzoic acid (generated by addition of the enolate to the azide) may form an ion pair with the cinchona alkaloid and, thus, lead to catalyst deactivation. Thus, we tested the addition of inorganic bases, but the yield could not be improved either (entry 8). Interestingly the reaction was significantly faster in the beginning (almost 50% conversion of the starting material after 90 min), but then stalled again, and the addition of additional amounts of azidation reagent **1** after that period did not increase the yield measurably (other bases were tested as well, but with the same outcome). The only way to improve the yield somewhat was by using a stoichiometric amount of amine **C2** (entries 9 and 10). By using a two-fold excess of the azide-reagent **1** we observed a slightly higher yield with an identical enantiomeric ratio (entry 11) but, again, this could not be improved by longer reaction times (entry 12) or by adding reagent **1** portion-wise. At this point we decided to change the nature of the azidation agent and used compound **2** instead. We reasoned that the hereby-formed benzylic alcohol reaction product is less likely to form an ion pair with the catalyst and should, thus, not result in catalyst deactivation. Surprisingly, however, even with this system no higher yield was possible and, in contrast to the use of **1**, the *e.r.* dropped significantly (entry 13). Using reagent **1** again and changing the solvent also had no positive effect on yield and/or *e.r.* (entries 14 and 15) and we, therefore, tested the other cinchona derivatives **C3** and **C4** next (entries 16 and 17), but neither of them matched the performance of **C2** (pseudoenantiomeric systems performed more or less equally selective in favor of the other enantiomer of **4a**). Accordingly, as we stuck to yields around 40–50% and an enantiomeric ratio of around 80:20 with catalyst **C1**, we finally tested a few other commonly-employed organocatalysts. Unfortunately however, neither Takemoto’s catalyst **C5** [31], nor Lambert’s base catalyst **C6** [32] or sparteine **C7** performed anywhere as well as cinchonidine (**C1**) (compare entry 11 with entries 18–20) and, thus, the result depicted in entry 11 (40% yield, *e.r.* = 80:20) was the best we could achieve by using a reasonably low amount of 20 mol% catalyst.

After testing the azidation with the hypervalent iodine reagents **1** and **2** we next focused on the reaction of β-ketoester **3a** with TMSN_3_ (**5**) under oxidative conditions in the presence of ammonium iodide catalysts (Table 2). First experiments using H_2_O_2_ and the chiral catalyst **C1** showed that this reaction can be carried out with good yield under these conditions (entry 1). It was also shown that the ammonium iodide is necessary for this reaction (entry 2). Unfortunately, however, no asymmetric induction could be achieved in these first experiments (entries 1 and 3). When changing the oxidant (entries 4 and 5) the reaction progress was similarly fast but, again, the product could only be accessed in almost racemic form and neither variations of the conditions nor of the chiral catalyst gave any better results. Despite this lack in selectivity we were attracted by the simplicity of this procedure and, thus, also quickly tested the easily-accessible achiral bifunctional ammonium iodide **C8**, which catalyzed the racemic azidation of **3a** very well under operationally simple conditions (entry 6).

After this broad screening of different catalysts and conditions for the α-azidation of β-ketoester **3a** under organocatalytic conditions we finally evaluated the substrate scope by testing a few different ketoesters **3** under the asymmetric conditions with cinchonidine (**C2**) and azide-reagent **1** (according to the conditions shown in entry 11 in Table 1), as well as using the racemic oxidative strategy (entry 6, Table 2).

**Scheme 3:**
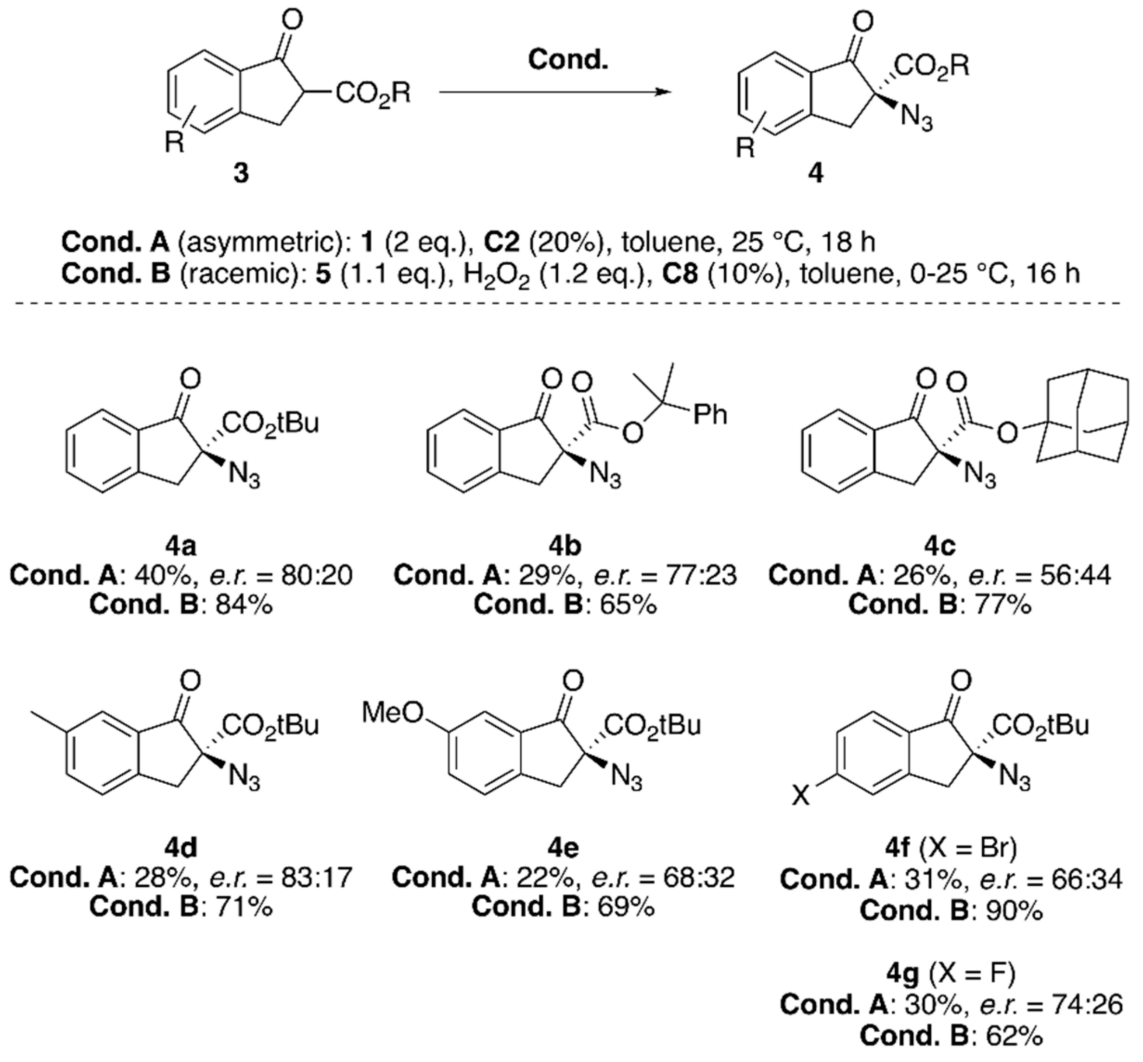
Application scope.

As can be seen in Scheme 3, in neither case the yield for the asymmetric reaction was higher than 40%, illustrating the general difficulties already observed in the initial screening. Somewhat surprisingly, the achieved enantioselectivity was rather strongly substituent influenced, i.e., the adamantyl-ester **4c** could be accessed with rather low selectivity only, which is in strong contrast to recent observations in other transformations [24,26]. On the other hand, the racemic protocol performed similarly well for all substrates without any further optimization, thus proving the generality for this reaction.

## 3. Experimental Section 

^1^H- and ^13^C-NMR spectra were recorded on a Bruker Avance III 300 MHz spectrometer and were referenced at the solvent peak. High-resolution mass spectra were obtained using a Thermo Fisher Scientific LTQ Orbitrap XL with an Ion Max API source. HPLC analyses were performed by using a Dionex Summit HPLC system with the chiral stationary phases indicated in the specific cases. All reactions were performed under an Ar atmosphere unless stated otherwise. Catalysts **C1**–**C7** were either commercial or prepared as described recently [32,35]. Starting β-ketoesters **3** were either purchased from commercial suppliers or prepared as described previously [26,27,28]. Hypervalent iodine-base reagents **1** and **2** were prepared as described recently [18,19,20]. Product **4** is a known compound and the analytical data match those reported previously [19,20,34]. Further experimental and analytical details (like NMR spectra and HPLC copies) can be found in the online supporting information.

**Catalyst C8:** To a solution of 3,5-bis(trifluoromethyl)phenyl isothiocyanate (0.336 mL, 1.84 mmol) in toluene (2 mL) was added *N*,*N*-dimethylethylenediamine (0.201 mL, 1.84 mmol). After stirring for 14 h at room temperature the reaction mixture was directly subjected to column chromatography (silica gel, CH_2_Cl_2_/MeOH, 20:1) to afford 625 mg (95%) of the known thiourea intermediate [36]. A mixture of this thiourea (100 mg, 0.278 mmol) and benzylbromide (95 mg, 0.556 mmol) was stirred in 2 mL of CH_3_CN for 15 h at room temperature. The reaction mixture was directly subjected to column chromatography (silica gel, CH_2_Cl_2_/MeOH, 10:1) to yield 148 mg (92%) of catalyst **C8**. ^1^H NMR (300 MHz, CDCl_3_) δ 9.83 (s, 1H), 8.72 (t, *J* = 5.7 Hz, 1H), 8.22 (s, 2H), 7.40–7.58 (m, 6H), 4.81 (s, 2H), 4.35–4.46 (m, 2H), 3.9 (t, *J* = 5.8 Hz, 2H), 3.26 (s, 6H). ^13^C NMR (75 MHz, CDCl_3_) δ 181.7, 140.3, 132.9, 131.4 (q, *J* = 34 Hz), 131.3, 125.9, 123.1 (q, *J* = 274 Hz), 123.0, 122.9, 117.9, 68.8, 63.5, 50.6, 38.2. HRMS-ESI): calcd for C_20_H_22_F_6_N_3_S^+^ (M)^+^ 450.1433, found 450.1439.

**Azidation Conditions A — General procedure:** The ketoester **3** (0.1 mmol) and catalyst **C2** (20 mol%) were dissolved in toluene (3 mL). Benziodoxole **1** (2 eq.) was added and the reaction mixture was stirred for 18–40 h (no difference in yield). The mixture was filtered over Na_2_SO_4_ and the solvent was evaporated and the product was purified by column chromatography (DCM/heptanes = 5/1, silica gel 60). 

**Azidation Conditions B — General procedure:** The ketoester **3** (0.1 mmol) and catalyst **C8** (10 mol%) were dissolved in toluene (3 mL) under argon at 0 °C. Then H_2_O_2_ (35%, 1.2 eq.) and TMSN_3_ (1.1 eq.) were added. The resulting mixture was stirred overnight. The mixture was filtered over Na_2_SO_4_ and the solvent was evaporated and the product was purified by column chromatography (DCM/heptanes = 5/1, silica gel 60). 

**Product 4a:** Obtained in the yields and with the selectivities given in Scheme 3 in the main manuscript. Analytical data match those reported recently [19,20,34]

^1^H-NMR (CDCl_3_, 300 MHz, 298 K), *δ*/ppm: 7.82 (d, *J* = 7.7 Hz, 1H), 7.66 (t, *J* = 7.4 Hz, 1H), 7.48–7.41 (m, 2H), 3.64 (d, *J* = 17.2 Hz, 1H), 2.99 (d, *J* = 17.2 Hz, 1H), 1.46 (s, 9H); ^13^C NMR (75 MHz, CDCl_3_, 298 K): *δ*/ppm = 198.1, 167.4, 152.3, 136.4, 133.3, 128.4, 126.5, 84.6, 70.6, 38.7, 28.0; MS (ESI+): *m/z* calcd for C_14_H_16_N_3_O_3_ [M + H]^+^: 274.12; found: 274.14. The enantioselectivity was determined by HPLC analysis (Chiralcel OJ-H, hexane/*i*-PrOH = 3/1, 1.0 mL min^−1^, 10 °C; *t*_R_ / min = 6.1 (major), 10.0 (minor).

**Product 4b:** Obtained in the yields and with the selectivities given in Scheme 3 in the main manuscript. Analytical data match those reported recently [19,20,34] 

^1^H-NMR (CDCl_3_, 300 MHz, 298 K), *δ*/ppm: 7.85 (d, *J* = 7.8 Hz, 1H), 7.68 (t, *J* = 7.5 Hz, 1H), 7.50–7.43 (m, 2H), 7.33–7.22 (m, 5H), 3.67 (d, *J* = 17.4 Hz, 1H), 3.02 (d, *J* = 17.3 Hz, 1H), 1.81 (s, 3H), 1.77 (s, 3H); ^13^C NMR (75 MHz, CDCl_3_, 298 K): *δ*/ppm = 197.9, 166.7, 152.2, 144.5, 136.4, 133.4, 128.5, 128.4, 127.6, 126.5, 125.7, 124.3, 85.5, 70.7, 38.6, 28.7, 28.1; HRMS (ESI+): m/z calcd for C_19_H_21_N_4_O_3_ [M + NH_4_]^+^: 353.1608; found: 353.1610. The enantioselectivity was determined by HPLC analysis (Chiralcel OJ-H, hexane/*i*-PrOH = 3/1, 1.0 mL min^−1^, 10 °C; tR/min = 11.3 (major), 15.4 (minor).

**Product 4c:** Obtained in the yields and with the selectivities given in Scheme 3 in the main manuscript. Analytical data match those reported recently [19,20,34] 

^1^H-NMR (CDCl_3_, 300 MHz, 298 K), *δ*/ppm: 7.81 (d, *J* = 7.6 Hz, 1H), 7.66 (t, *J* = 7.5 Hz, 1H), 7.48–7.40 (m, 2H), 3.64 (d, *J* = 17.2 Hz, 1H), 2.98 (d, *J* = 17.3 Hz, 1H), 2.15 (s, 3H), 2.08 (s, 6H), 1.63 (s, 6H); ^13^C NMR (75 MHz, CDCl_3_, 298 K): δ/ppm = 198.2, 167.1, 152.4, 136.3, 133.4, 128.4, 126.4, 125.6, 84.7, 70.6, 41.3, 38.7, 36.1, 31.0; MS (ESI+): m/z calcd for C_20_H_25_N_4_O_3_ [M + NH_4_]^+^: 369.19; found: 369.20. The enantioselectivity was determined by HPLC analysis (YMC CHIRALART Cellulose-SB, hexane/*i*-PrOH = 300/1, 1.0 mL min^−1^, 10 °C; tR/min = 9.1 (minor), 12.1 (major).

**Product 4d:** Obtained in the yields and with the selectivities given in Scheme 3 in the main manuscript. Analytical data match those reported recently [19,20,34] 

^1^H-NMR (CDCl_3_, 300 MHz, 298 K), *δ*/ppm: 7.65 (d, *J* = 7.5 Hz, 1H), 7.47 (d, *J* = 7.3 Hz, 1H), 7.35 (t, *J* = 7.4 Hz, 1H), 3.52 (d, *J* = 17.3 Hz, 1H) , 2.87 (d, *J* = 17.3 Hz, 1H), 2.34 (s, 3H), 1.47 (s, 9H); ^13^C NMR (75 MHz, CDCl_3_, 298 K): *δ*/ppm = 198.3, 167.6, 151.3, 136.9, 135.8, 133.1, 128.6, 123.0, 84.6, 70.5, 37.6, 28.0, 17.9; HRMS (ESI+): *m/z* calcd for C_15_H_18_N_3_O_3_ [M + H]^+^: 288.1343; found: 288.1345. The enantioselectivity was determined by HPLC analysis (Chiralcel OD-H, hexane/*i*-PrOH = 300/1, 1.0 mLmin^−1^, 10 °C; *t*_R_/min = 9.9 (minor), 13.2 (major).

**Product 4e:** Obtained in the yields and with the selectivities given in Scheme 3 in the main manuscript. Analytical data match those reported recently [19,20,34] 

^1^H-NMR (CDCl_3_, 300 MHz, 298 K), *δ*/ppm: 7.34 (d, *J* = 8.1 Hz, 1H), 7.28–7.20 (m, 2H), 3.85 (s, 3H), 3.55 (d, *J* = 16.8 Hz, 1H) , 2.91 (d, *J* = 16.9 Hz, 1H), 1.46 (s, 9H); ^13^C NMR (75 MHz, CDCl_3_, 298 K): *δ*/ppm = 198.0, 167.5, 160.1, 145.3, 134.5, 127.2, 125.9, 106.5, 84.6, 71.3, 55.8, 38.1, 28.0; HRMS (ESI+): *m/z* calcd for C_15_H_18_N_3_O_4_ [M + H]^+^: 304.1292; found: 304.1293. The enantioselectivity was determined by HPLC analysis (Chiralcel OD-H, hexane/*i*-PrOH = 300/1, 1.0 mL min^−1^, 10 °C; *t*_R_/min = 8.6 (minor), 10.0 (major).

**Product 4f:** Obtained in the yields and with the selectivities given in Scheme 3 in the main manuscript. Analytical data match those reported recently [19,20,34]

^1^H-NMR (CDCl_3_, 300 MHz, 298 K), *δ*/ppm: 7.70–7.63 (m, 2H), 7.58 (d, *J* = 8.1 Hz, 1H), 3.61 (d, *J* = 17.5 Hz, 1H), 2.96 (d, *J* = 17.4 Hz, 1H), 1.46 (s, 9H); ^13^C NMR (75 MHz, CDCl_3_, 298 K): *δ*/ppm = 197.0, 167.0, 153.8, 132.2, 132.1, 132.0, 129.8, 126.7, 84.9, 70.5, 38.3, 28.0; MS (ESI+): *m/z* calcd for C_14_H_15_BrN_3_O_3_ [M + H]^+^: 352.03; found: 352.04. The enantioselectivity was determined by HPLC analysis (Chiralpak AD-H, hexane/*i*-PrOH = 100/1, 0.5 mL min^−1^, 10 °C; *t*_R_/min = 19.7 (major), 22.5 (minor).

**Product 4g:** Obtained in the yields and with the selectivities given in Scheme 3 in the main manuscript. Analytical data match those reported recently [19,20,34] 

^1^H-NMR (CDCl_3_, 300 MHz, 298 K), *δ*/ppm: 7.87–7.80 (m, 1H), 7.14 (t, *J* = 8.5 Hz, 1H), 7.35 (t, *J* = 7.4 Hz, 1H), 3.62 (d, *J* = 17.4 Hz, 1H) , 2.97 (d, *J* = 17.6 Hz, 1H), 1.46 (s, 9H); ^13^C NMR (75 MHz, CDCl_3_, 298 K): *δ*/ppm = 196.3, 167.2, 168.1, 155.4, 129.7, 128.1, 116.9, 113.4, 84.9, 70.7, 38.5, 28.0; HRMS (ESI+): *m/z* calcd for C_14_H_15_FN_3_O_3_ [M + H]^+^: 292.1092; found: 292.1093. The enantioselectivity was determined by HPLC analysis (Chiralcel OJ-H, hexane/*i*-PrOH = 3/1, 1.0 mL min^−1^, 10 °C; *t*_R_/min = 6.2 (major), 9.0 (minor).

## 4. Conclusions

In conclusion, we have carried out detailed investigations concerning the organocatalytic (asymmetric) **α**-azidation of prochiral **β**-ketoesters. It was shown that, in general, an asymmetric procedure for such a reaction is possible by using simple cinchona alkaloid catalysts. Unfortunately, however, these reactions were relatively low yielding and only modestly enantioselective (up to *e.r.* = 83:17). On the other hand, a very simple racemic version for this α-azidation was developed by using TMSN_3_ as the azide-source under oxidative conditions with quaternary ammonium iodides as catalysts.

## Figures and Tables

**Table 1:**
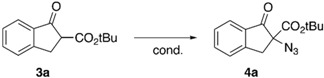
Screening and optimization of the α-azidation of **3a** with hypervalent iodine-transfer reagents.

Entry ^1^	Cat.	N_3_^+^	Base	Solv.	T (°C)	t (h)	Yield ^2^ (%)	*e.r.* (S:R) ^3^
1	**C1** (10%)	**1** (1.1 eq.)	K_2_CO_3_ (1.1 eq.)	toluene	25	3	80	56:44
2	**C1** (10%)	**1** (1.1 eq.)	-	toluene	25	16	50	55:45
3	**C1** (10%)	**1** (1.1 eq.)	K_2_CO_3_ (1.1 eq.)	MTBE	25	0.5	85	55:45
4	**C1** (10%)	**1** (1.1 eq.)	DMAP (1.1 eq.)	MTBE	25	0.5	88	52:48
5	**C2** (20%)	**1** (1.1 eq.)	-	toluene	25	18	18	65:35
6	**C2** (40%)	**1** (1.1 eq.)	-	toluene	25	40	44	80:20
7	**C2** (40%)	**1** (1.1 eq.)	-	toluene	50	40	39	67:33
8	**C2** (40%)	**1** (1.1 eq.)	K_2_CO_3_ (1.1 eq.)	toluene	25	1.5	40	77:23
9	**C2** (100%)	**1** (1.1 eq.)	-	toluene	25	1.5	52	81:19
10	**C2** (100%)	**1** (1.1 eq.)	-	MTBE	25	1.5	66	67:33
11	**C2** (20%)	**1** (2 eq.)	-	toluene	25	18	40	80:20
12	**C2** (20%)	**1** (2 eq.)	-	toluene	25	72	41	80:20
13	**C2** (20%)	**2** (2 eq.)	-	toluene	25	18	24	56:44
14	**C2** (20%)	**1** (2 eq.)	-	CH_2_Cl_2_	25	18	39	75:25
15	**C2** (20%)	**1** (2 eq.)	-	THF	25	18	19	55:45
16	**C3** (20%)	**1** (2 eq.)	-	toluene	25	18	35	63:37
17	**C4** (20%)	**1** (2 eq.)	-	toluene	25	18	15	58:42
18	**C5** (20%)	**1** (2 eq.)	-	toluene	25	18	traces	n.d.
19	**C6** (20%)	**1** (2 eq.)	-	toluene	25	18	7	54:46
20	**C7** (20%)	**1** (2 eq.)	-	toluene	25	18	28	46:54

^1^ All reactions were carried out on a 0.1 mmol scale; ^2^ Isolated yield; ^3^ Determined by HPLC using a chiral stationary phase and assigned in analogy to the reported retention times [20,34].

**Table 2:**
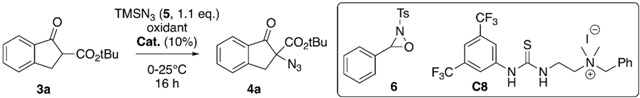
α-Azidation of **3a** under oxidative conditions using TMSN_3_ (**5**).

Entry	Oxidant	Solvent	Cat.	Yield [%] ^1^	*e.r.* [S:R] ^2^
1	H_2_O_2_ (35%) (1.2 eq.)	toluene	**C1**	81	55:45
2	H_2_O_2_ (35%) (1.2 eq.)	toluene	-	0	-
3	H_2_O_2_ (35%) (1.2 eq.)	AcN	**C1**	86	53:47
4	t-BuOOH (1.2 eq.)	toluene	**C1**	76	54:46
5	**6** (1.2 eq.)	toluene	**C1**	80	52:48
6	H_2_O_2_ (35%) (1.2 eq.)	toluene	**C8**	84	-

^1^ Isolated yield; ^2^ Determined by HPLC using a chiral stationary phase and assigned in analogy to the reported retention times [20,34].

## Supplementary Materials

NMR spectra and HPLC traces are available online.

## References

[1] Bräse S., Banert K. (2009). Organic Azides: Syntheses and Applications.

[2] Bräse S., Gil C., Knepper K., Zimmermann V. (2005). Organic Azides: An Exploding Diversity of a Unique Class of Compounds. Angew. Chem. Int. Ed..

[3] Bock V.D., Hiemstra H., van Maarseveen J.H. (2006). Cu^I^-Catalyzed Alkyne–Azide “Click” Cycloadditions from a Mechanistic and Synthetic Perspective. Eur. J. Org. Chem..

[4] Liang L., Astruc D. (2011). The Copper(I)-Catalyzed Alkyne-Azide Cycloaddition (CuAAC) “Click” Reaction and its Applications. An Overview. Coord. Chem. Rev..

[5] Singh M.S., Chowdhury S., Koley S. (2016). Advances of Azide-Akyne Cycloaddition-Click Chemistry Over the Recent Decade. Tetrahedron.

[6] Gololobov Y.G., Kasukhin L.F. (1992). Recent Advances in the Staudinger Reaction. Tetrahedron.

[7] Scriven E.F.V., Turnbull K. (1988). Azides: Their Preparation and Synthetic Uses. Chem. Rev..

[8] Huang D., Yan G. (2017). Recent Advances in Reactions of Azides. Adv. Synth. Catal..

[9] Goswami M., de Bruin B. (2017). Metal-Catalyed Azidation of Organic Molecules. Eur. J. Org. Chem..

[10] Ding P.-G., Hu X.-S., Zhou F., Zhou J. (2018). Catalytic Enantioselective Synthesis of a-Chiral Azides. Org. Chem. Front..

[11] Phan T.B., Mayr H. (2006). Nucleophilic Reactivity of the Azide Ion in Various Solvents. J. Phys. Org. Chem..

[12] Harschneck T., Hummel S., Kirsch S.F., Klahn P. (2012). Practical Azidation of 1,3-Dicarbonyls. Chem. Eur. J..

[13] Galligan M.J., Akula R., Ibrahim H. (2014). Unified Strategy for Iodine(III)-Mediated Halogenation and Azidation of 1,3-Dicarbonyl Compounds. Org. Lett..

[14] Dhineshkumar J., Prabhu K.R. (2016). An Efficient Tertiary Azidation of 1,3-Dicarbonyl Compounds in Water Catalyzed by Tetrabutylammonium Iodide. Eur. J. Org. Chem..

[15] Yasui K., Kojima K., Kato T., Odagi M., Kato M., Nagasawa K. (2016). Guanidinium iodide/urea hydrogen peroxide-catalyzed azidation of β-dicarbonyl compounds with trimethylsilyl azide. Tetrahedron.

[16] Zhdankin V. (2013). Hypervalent Iodine Chemistry, Preparation, Structure, and Synthetic Applications of Polyvalent Iodine Compounds.

[17] Yoshimura A., Zhdankin V. (2016). Advances in synthetic applications of hypervalent iodine compounds. Chem. Rev..

[18] Zhdankin V.V., Krasutsky A.P., Kuehl C.J., Simonsen A.J., Woodward J.K., Mismash B., Bolz J.T. (1996). Preparation, X-ray Crystal Structure, and Chemistry of Stable Azidoiodinaness Derivatives of Benziodoxole. J. Am. Chem. Soc..

[19] Vita M.V., Waser J. (2013). Azidation of β-Keto Esters and Silyl Enol Ethers with a Benziodoxole Reagent. Org. Lett..

[20] Deng Q.-H., Bleith T., Wadepohl H., Gade L.H. (2013). Enantioselective Iron-Catalyzed Azidation of b-Ketoesters and Oxindoles. J. Am. Chem. Soc..

[21] Wu X., Shirakawa S., Maruoka K. (2014). Efficient asymmetric synthesis of spiro-2(3H)-furanones via phase-transfer-catalyzed alkynylation. Org. Biomol. Chem..

[22] Fernandez Gonzalez D., Brand J.P., Mondiere R., Waser J. (2013). Ethynylbenziodoxolones (EBX) as Reagents for the Ethynylation of Stabilized Enolates. Adv. Synth. Catal..

[23] Chen M., Huang Z.-T., Zheng Q.-Y. (2015). Organic base-promoted enantioselective electrophilic cyanation of β-keto esters by using chiral phase-transfer catalysts. Org. Biomol. Chem..

[24] Chowdhury R., Schörgenhumer J., Novacek J., Waser M. (2015). Towards an asymmetric organocatalytic α-cyanation of β-ketoesters. Tetrahedron Lett..

[25] Schörgenhumer J., Tiffner M., Waser M. (2017). Chiral phase-transfer catalysis in the asymmetric α-heterofunctionalization of prochiral nucleophiles. Beilstein J. Org. Chem..

[26] Novacek J., Waser M. (2014). Syntheses and Applications of (Thio)Urea-Containing Chiral Quaternary Ammonium Salt Catalysts. Eur. J. Org. Chem..

[27] Novacek J., Monkowius U., Himmelsbach M., Waser M. (2016). Asymmetric α-chlorination of β-ketoesters using bifunctional ammonium salt catalysis. Monatsh. Chem..

[28] Novacek J., Izzo J.A., Vetticatt M.J., Waser M. (2016). Bifunctional Ammonium Salt Catalyzed Asymmetric α-Hydroxylation of β-Ketoesters by Simultaneous Resolution of Oxaziridines. Chem. Eur. J..

[29] Shirakawa S., Maruoka K. (2013). Recent Developments in Asymmetric Phase-Transfer Reactions. Angew. Chem. Int. Ed..

[30] Song C.E. (2009). Cinchona Alkaloids in Synthesis & Catalysis.

[31] Okino T., Hoashi Y., Takemoto Y. (2003). Enantioselective Michael Reaction of Malonates to Nitroolefins Catalyzed by Bifunctional Organocatalysts. J. Am. Chem. Soc..

[32] Bandar J.S., Lambert T.H. (2012). Enantioselective Brønsted Base Catalysis with Chiral Cyclopropenimines. J. Am. Chem. Soc..

[33] Uyanik M., Okamoto H., Yasui T., Ishihara K. (2010). Quaternary Ammonium (Hypo)iodite Catalysis for Enantioselective Oxidative Cycloetherification. Science.

[34] Shibatomi K., Soga Y., Narayama A., Fujisawa I., Iwasa S. (2012). Highly Enantioselective Chlorination of β-Keto Esters and Subsequent S_N_2 Displacement of Tertiary Chlorides: A Flexible Method for the Construction of Quaternary Stereogenic Centers. J. Am. Chem. Soc..

[35] Tiffner M., Novacek J., Busillo A., Gratzer K., Massa A., Waser M. (2015). Design of chiral urea-quaternary ammonium salt hybrid catalysts for asymmetric reactions of glycine Schiff bases. RSC Adv..

[36] Varga S., Jakab G., Drahos L., Holczbauer T., Czugler M., Soos T. (2011). Double Diastereocontrol in Bifunctional Thiourea Organocatalysis: Iterative Michael–Michael–Henry Sequence Regulated by the Configuration of Chiral Catalysts. Org. Lett..

