# Supplementary material for: Towards an Asymmetric Organocatalytic α-Azidation of β-Ketoesters
Source: Molecules. 2018 May 11;23(5):1142. doi: 10.3390/molecules23051142 (PMC6100502; doi:10.3390/molecules23051142)
Supplement: Supplementary file 1 [file molecules-23-01142-s001.pdf]

SUPPORTING INFORMATION

Towards an Asymmetric Organocatalytic α-Azidation of β-Ketoesters

Maximilian Tiffner, Lotte Stockhammer, Johannes Schörgenhumer, Katharina Röser, and Mario Waser*

Institute of Organic Chemistry, Johannes Kepler University Linz, Altenbergerstraße 69, 4040 Linz, Austria. Fax: +43 732 2468 5402; Tel: +43 732 2468 5411;

E-mail: Mario.waser@jku.at

1. Selected Copies of NMR Spectra 2
2. Chromatograms 8

1. Selected Copies of NMR Spectra

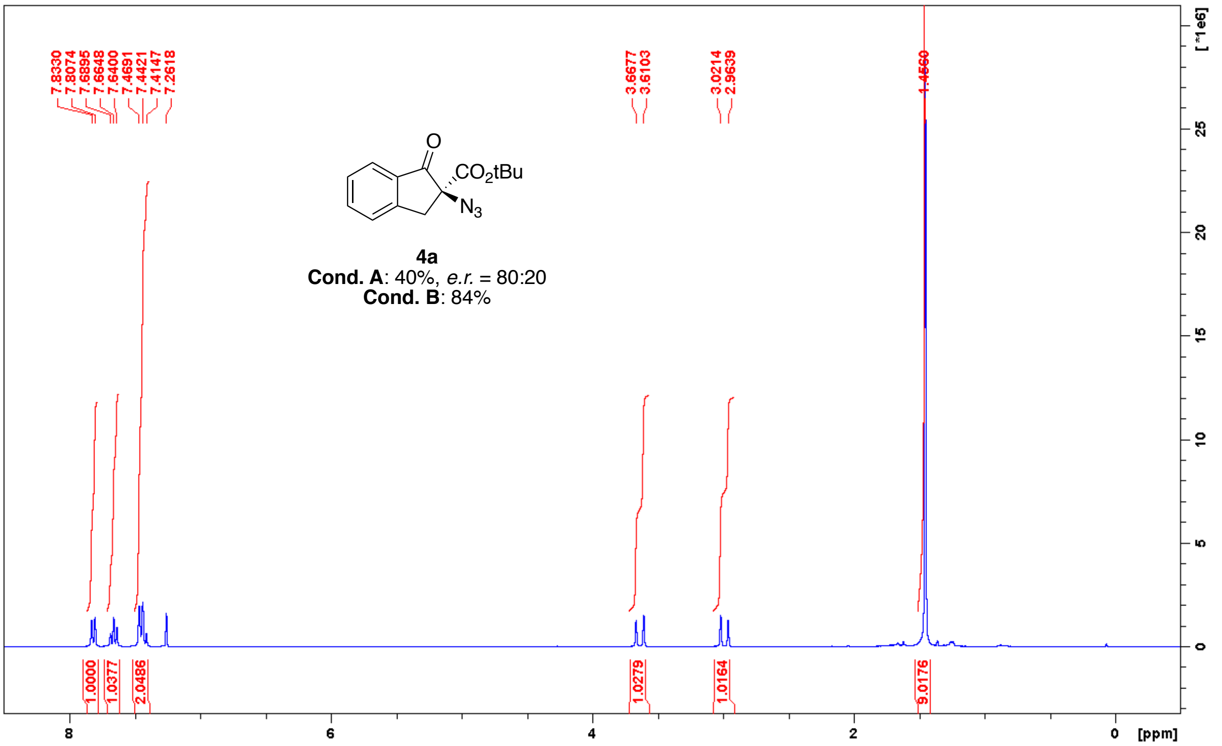

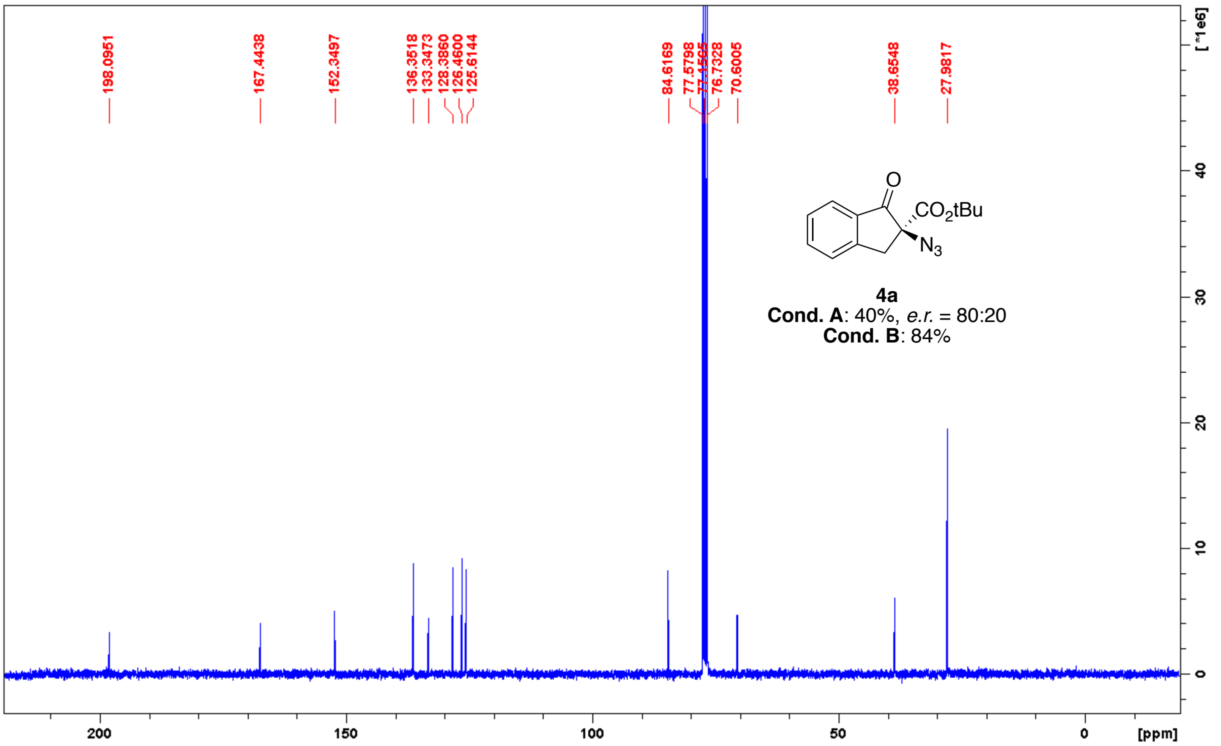

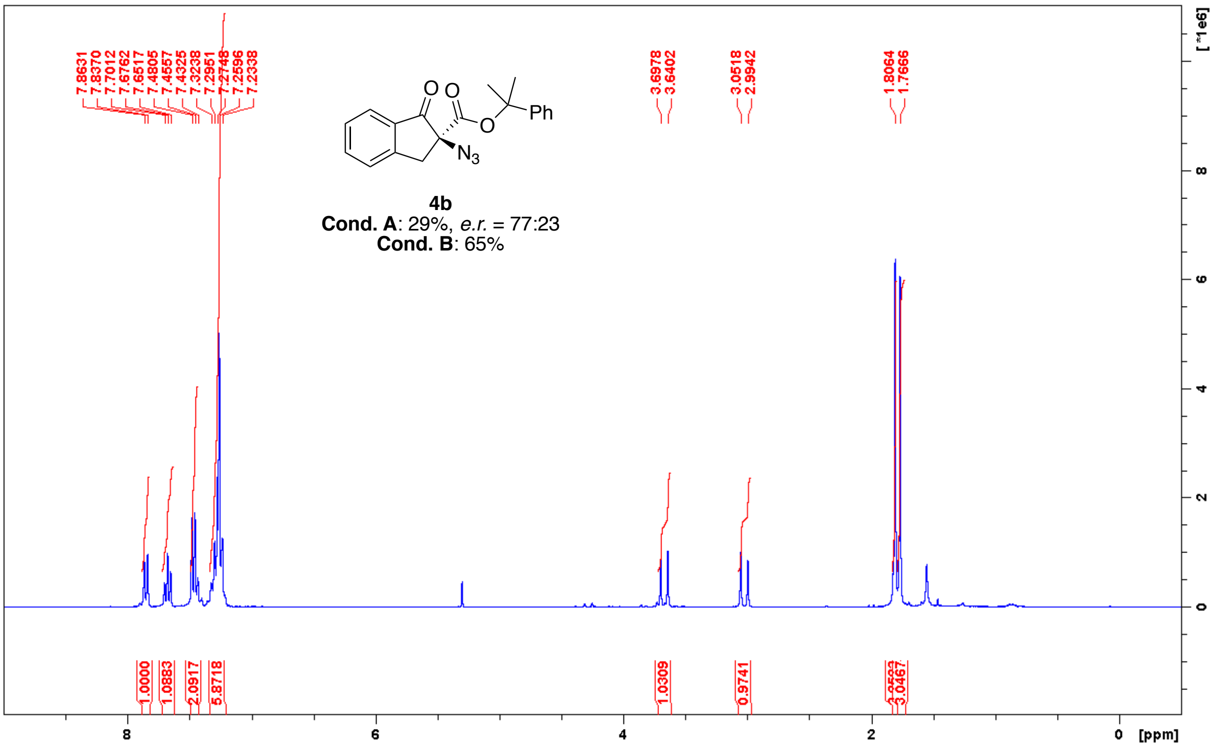
7.8631 7.8370 7.7012 7.6762 7.6517 7.4805 7.4557 7.4325 7.3238 7.2951 7.2748 7.2596 7.2338
3.6978 3.6402
3.0518 2.9942
1.8064 1.7666
O O
O
Ph
N3
4b
Cond. A: 29%, e.r. = 77:23
Cond. B: 65%
1.0000 1.0883 2.0917 5.8718
1.0309
0.9741
3.0467
[*1e6]
[ppm]

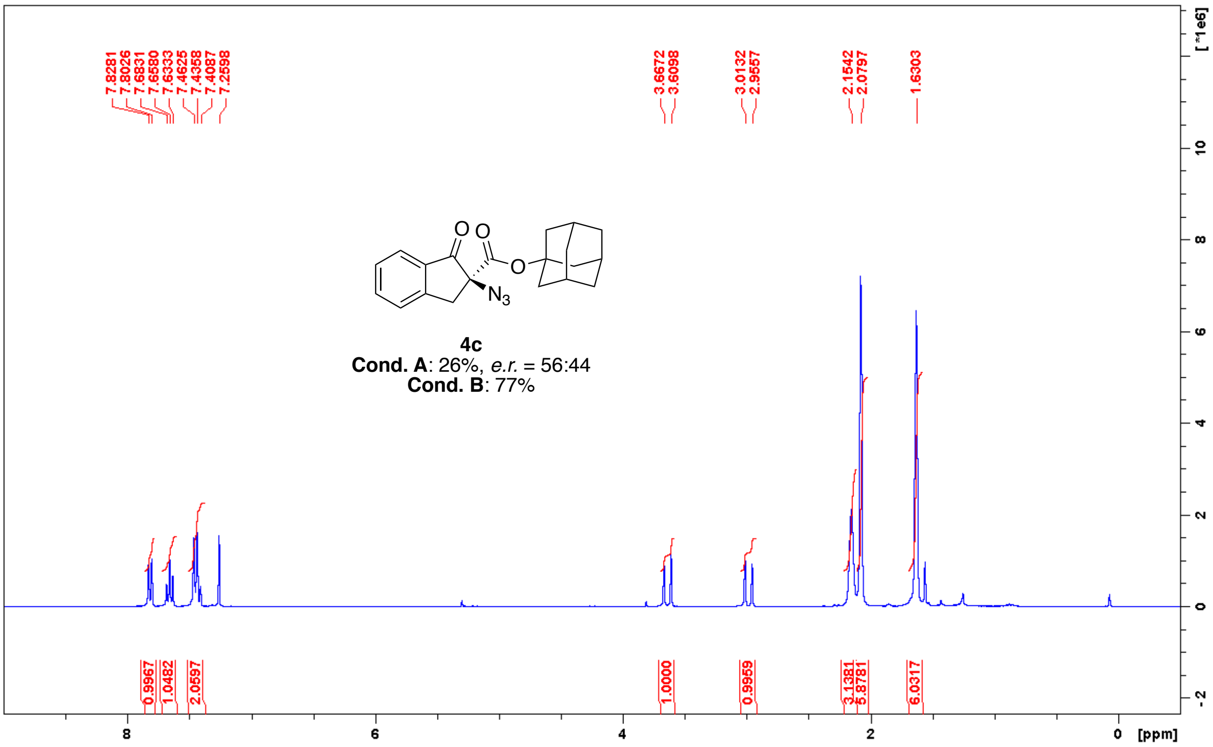
7.8281 7.8026 7.6831 7.6580 7.6333 7.4625 7.4358 7.4087 7.2598
3.6672 3.6098
3.0132 2.9557
2.1542 2.0797
1.6303
O O
O
N3
4c
Cond. A: 26%, e.r. = 56:44
Cond. B: 77%
0.9967 1.0482 2.0597
1.0000
0.9959
3.1381 5.8781
6.0317
[*1e6]
[ppm]

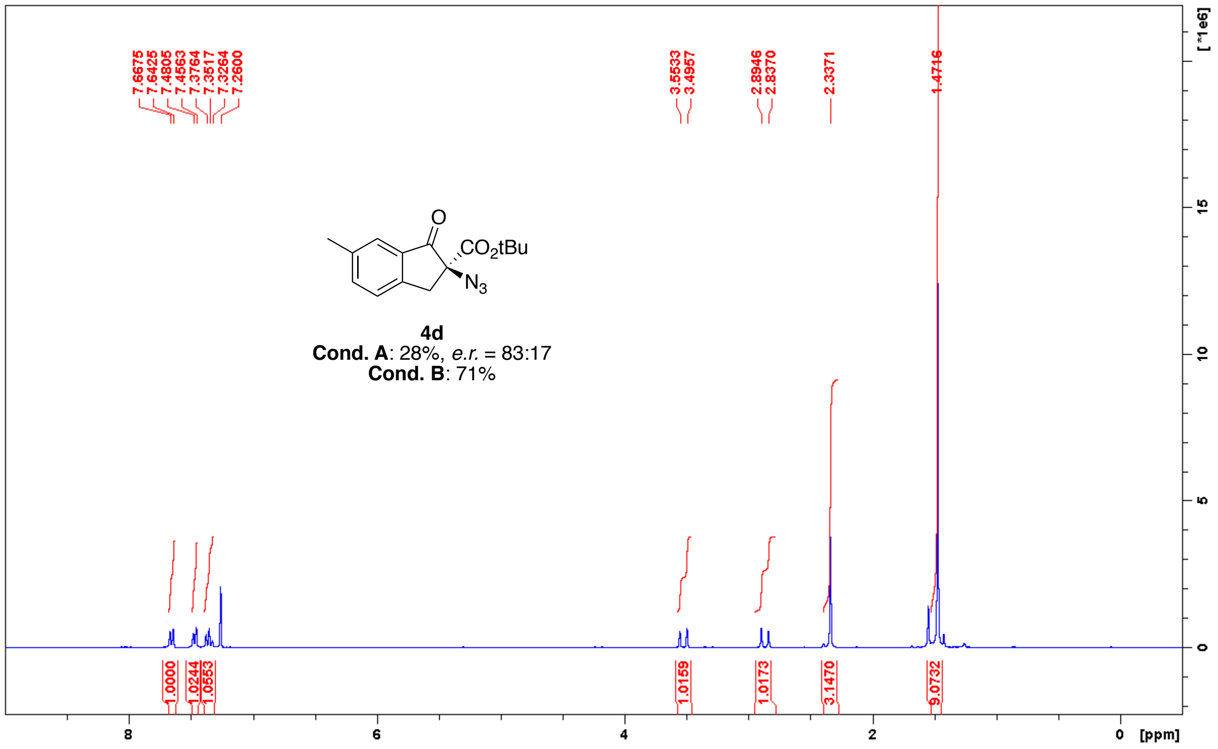

7.6675
7.6425
7.4805
7.4563
7.3764
7.3517
7.3264
7.2600
3.5533
3.4957
2.8946
2.8370
2.3371
1.4716
4d
Cond. A: 28%, e.r. = 83:17
Cond. B: 71%
1.0000
1.0244
1.0553
1.0159
1.0173
3.1470
9.0732
[ppm]
[*1e6]

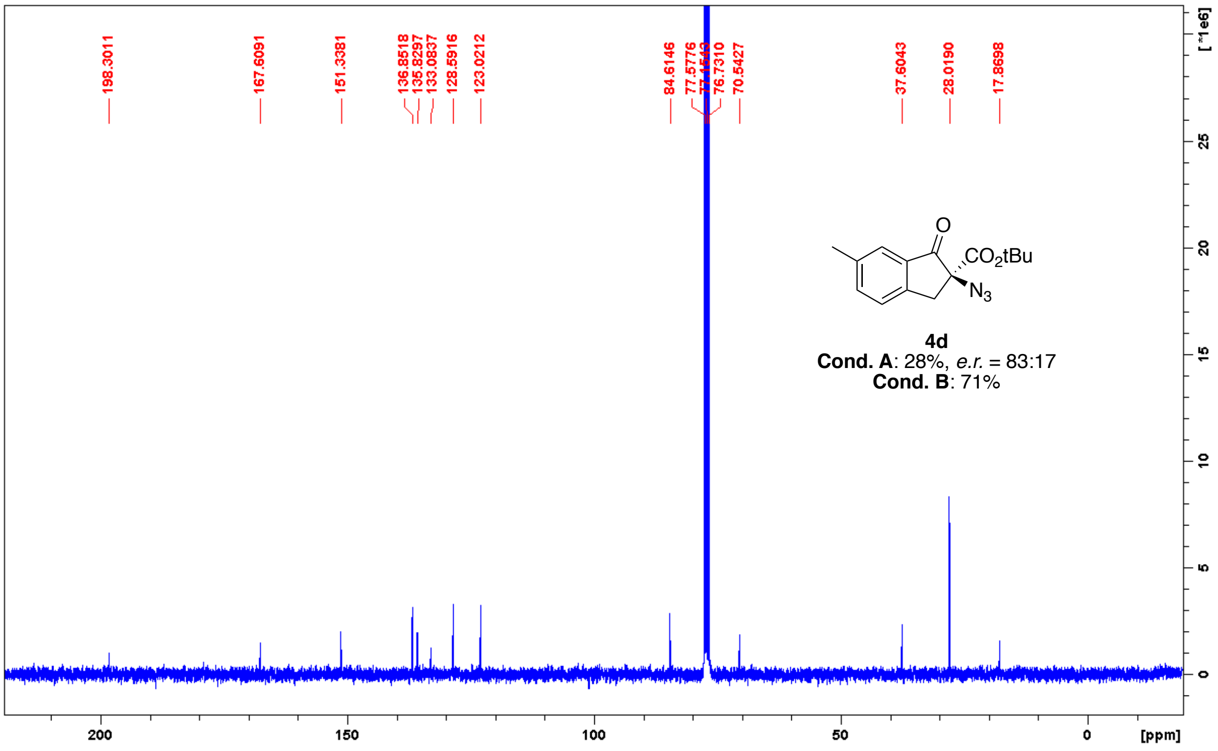

198.3011
167.6091
151.3381
136.8518
135.8297
133.0837
128.5916
123.0212
84.6146
77.5776
76.7310
70.5427
37.6043
28.0190
17.8698
4d
Cond. A: 28%, e.r. = 83:17
Cond. B: 71%
[ppm]
[*1e6]

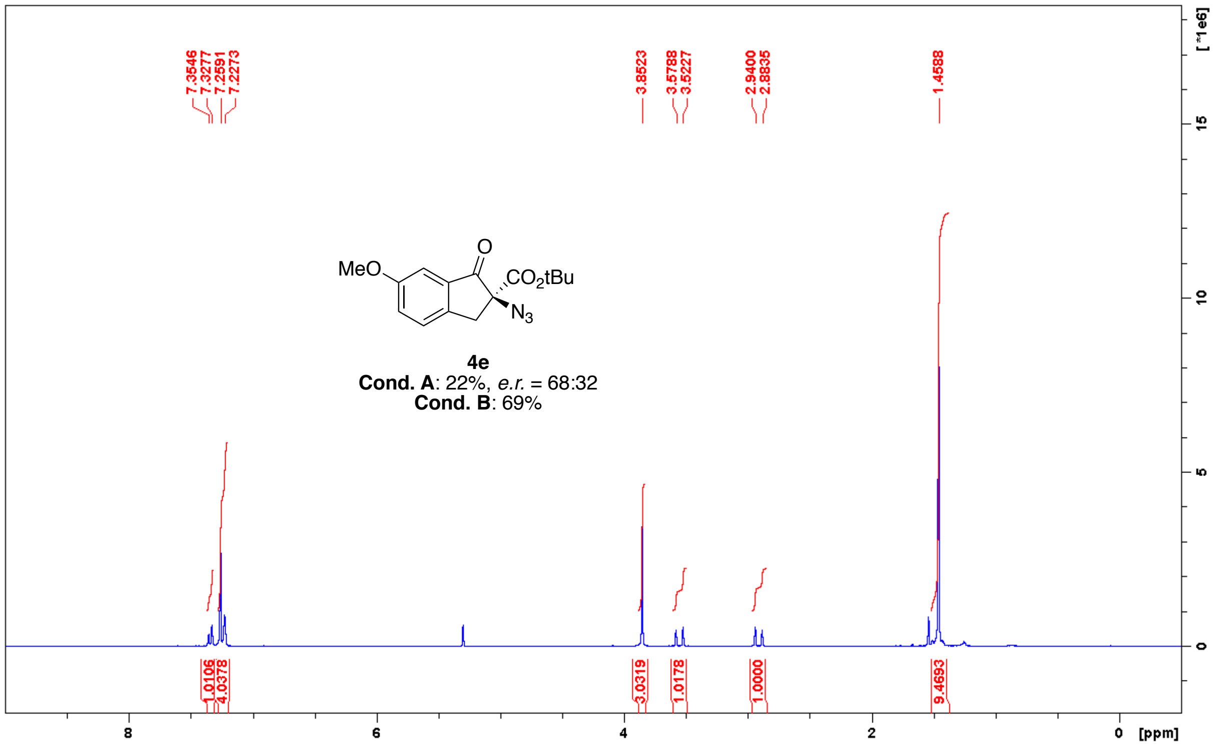
7.3546
7.3277
7.2591
7.2273
3.8523
3.5788
3.5227
2.9400
2.8835
1.4588
MeO
O
CO2tBu
N3
4e
Cond. A: 22%, e.r. = 68:32
Cond. B: 69%
1.0106
4.0378
3.0319
1.0178
1.0000
9.4693
[ppm]
[*1e6]

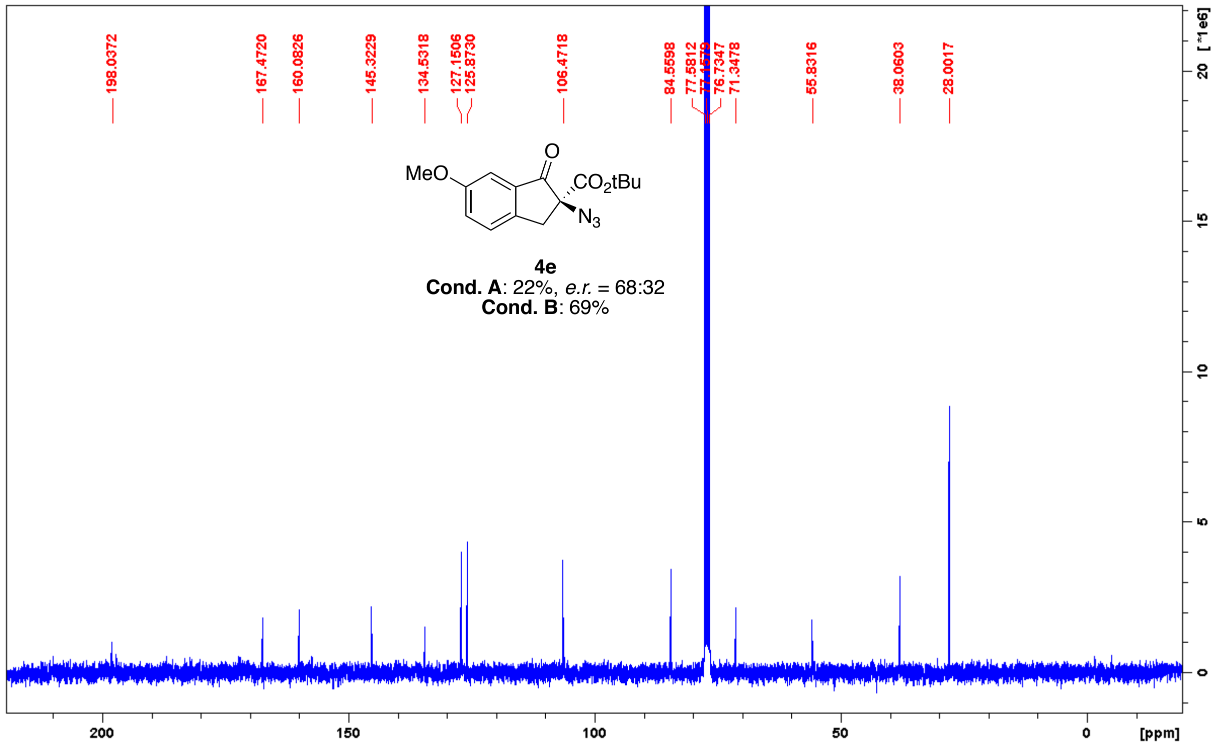
198.0372
167.4720
160.0826
145.3229
134.5318
127.1506
125.8730
106.4718
84.5598
77.5812
76.7347
71.3478
55.8316
38.0603
28.0017
MeO
O
CO2tBu
N3
4e
Cond. A: 22%, e.r. = 68:32
Cond. B: 69%
[ppm]
[*1e6]

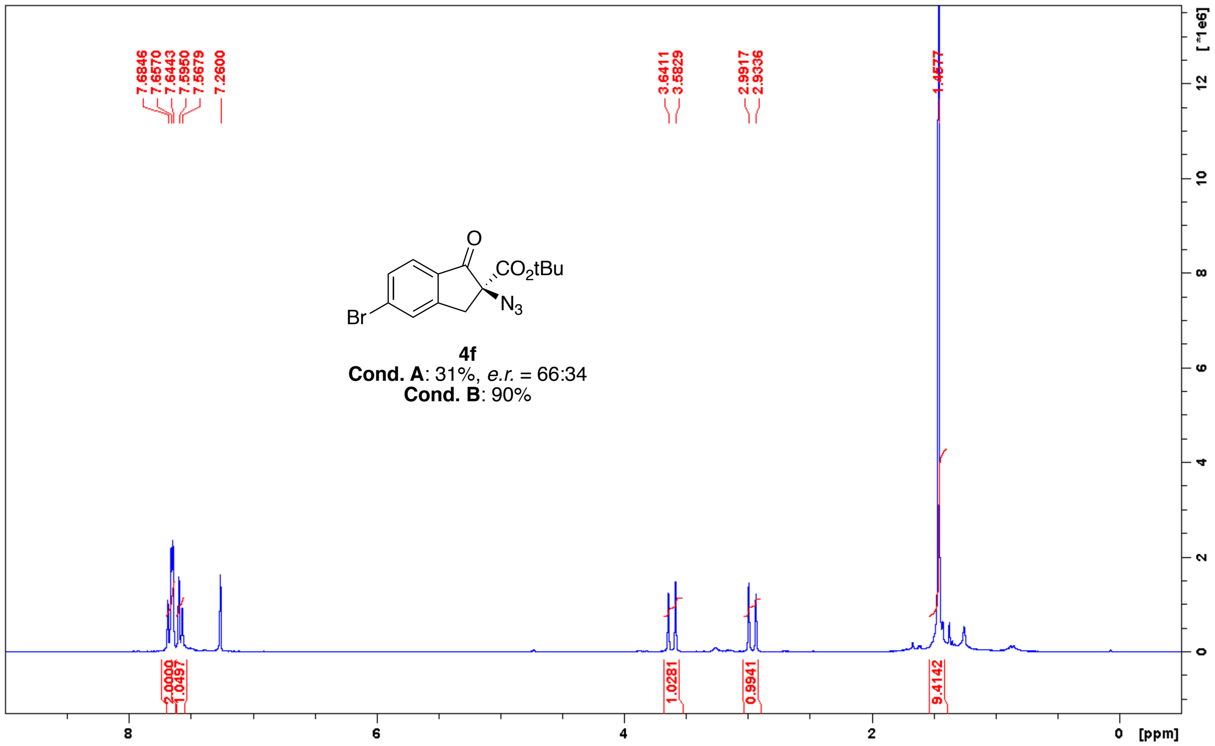
7.6846
7.6570
7.6443
7.5950
7.5679
7.2600
3.6411
3.5829
2.9917
2.9336
1.4577
O
CO2tBu
N3
Br
4f
Cond. A: 31%, e.r. = 66:34
Cond. B: 90%
2.0000
1.0497
1.0281
0.9941
9.4142
[*1e6]
[ppm]

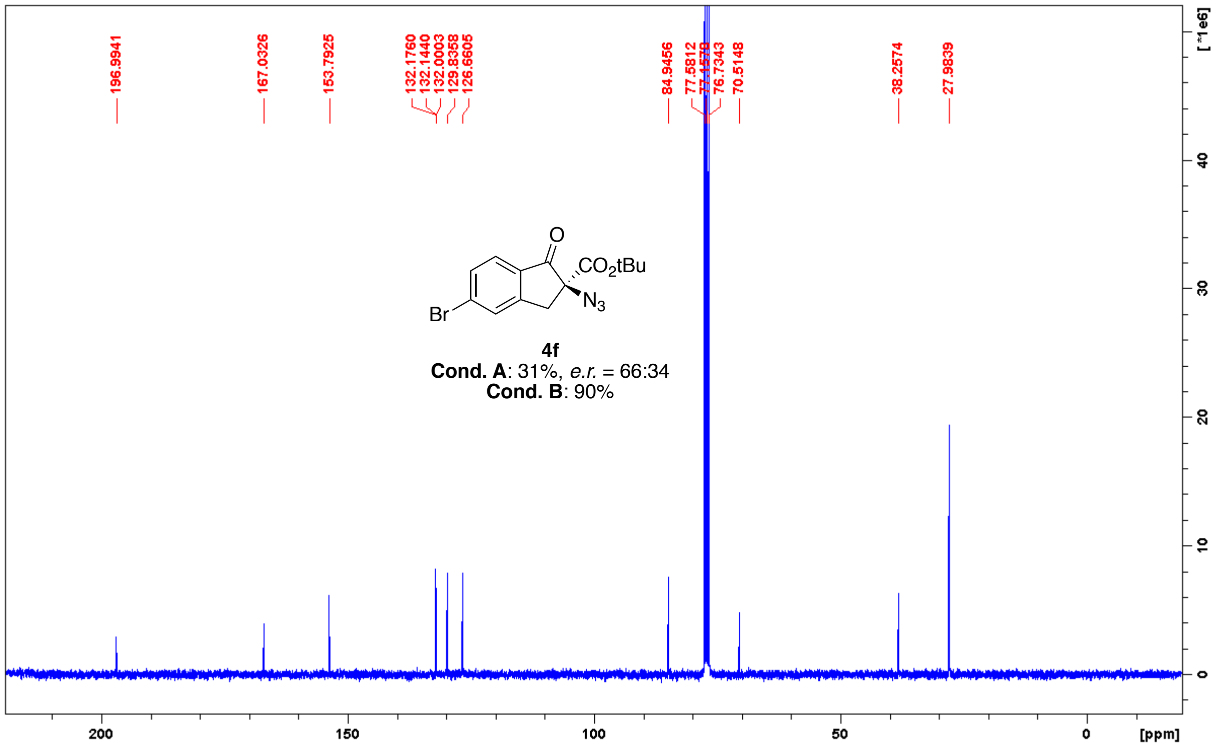
196.9941
167.0326
153.7925
132.1760
132.1440
132.0003
129.8358
126.6605
84.9456
77.5812
76.7343
70.5148
38.2574
27.9839
O
CO2tBu
N3
Br
4f
Cond. A: 31%, e.r. = 66:34
Cond. B: 90%
[*1e6]
[ppm]

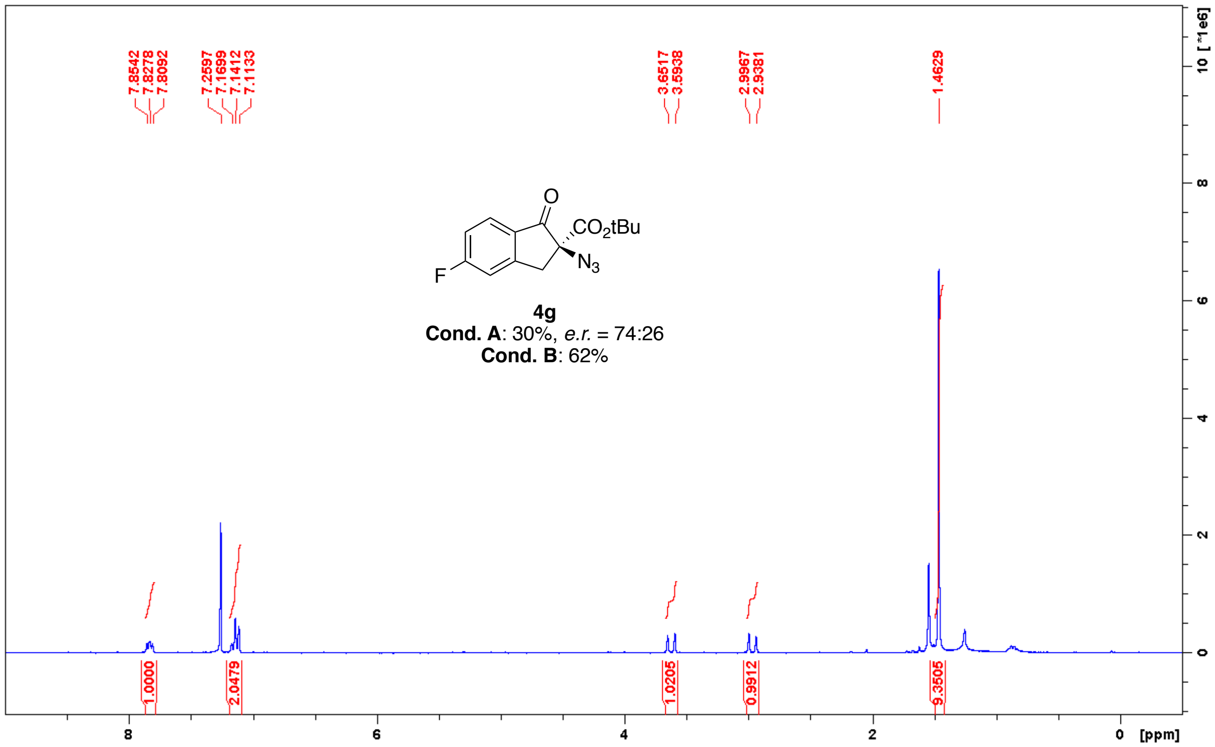
7.8542
7.8278
7.8092
7.2597
7.1699
7.1412
7.1133
3.6517
3.5938
2.9967
2.9381
1.4629
O
CO2tBu
N3
F
4g
Cond. A: 30%, e.r. = 74:26
Cond. B: 62%
1.0000
2.0479
1.0205
0.9912
9.3505
8
6
4
2
0
[ppm]
10 [*1e6]

2. Chromatograms

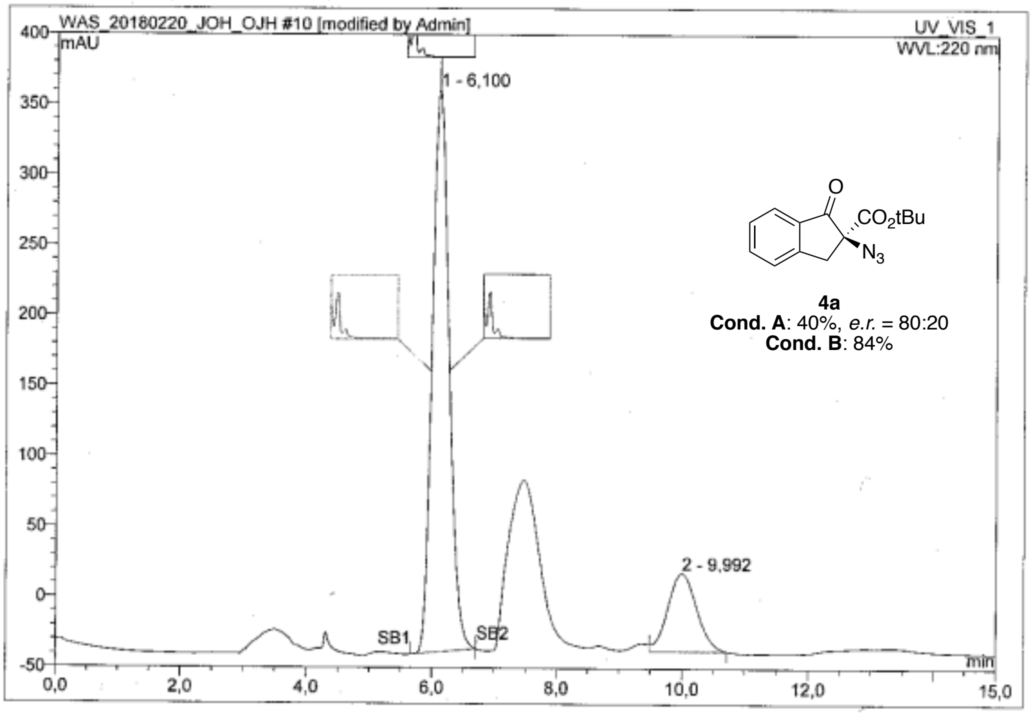

No.	Ret.Time min	Peak Name	Height mAU	Area mAU*min	Rel.Area %	Amount	Type
1	6,10	n.a.	399,543	124,843	80,72	n.a.	BMB*
2	9,99	n.a.	55,734	29,825	19,28	n.a.	MB*
Total:			455,277	154,668	100,00	0,000	

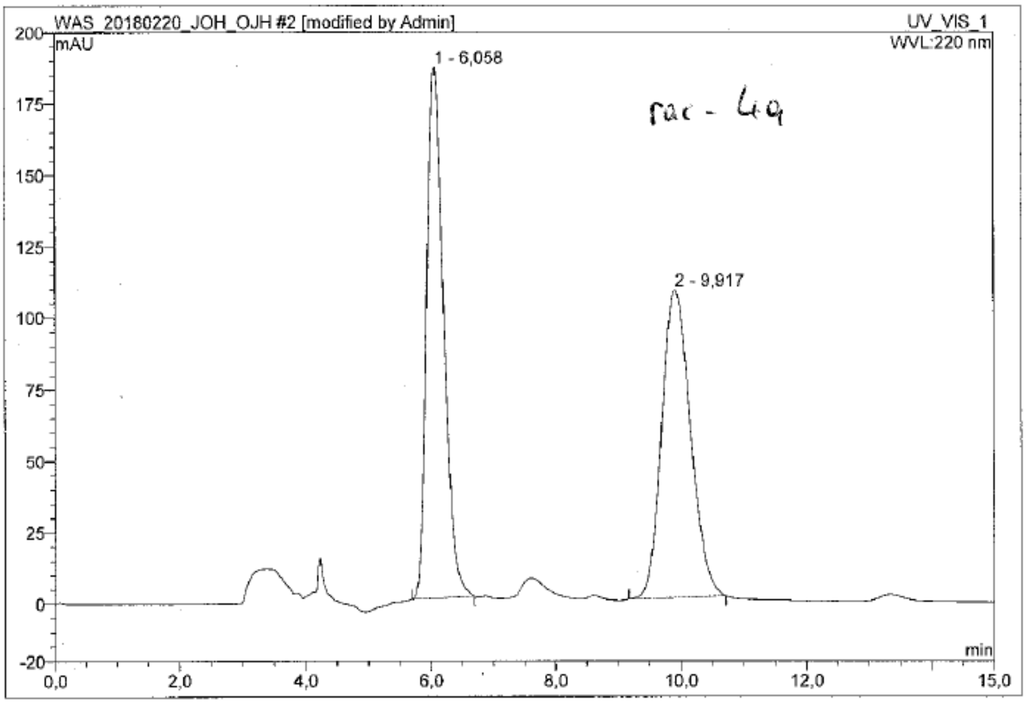

No.	Ret.Time min	Peak Name	Height mAU	Area mAU*min	Rel.Area %	Amount	Type
1	6,06	n.a.	185,868	58,480	50,49	n.a.	BMB*
2	9,92	n.a.	107,613	57,346	49,51	n.a.	BMB*
Total:			293,480	115,826	100,00	0,000	

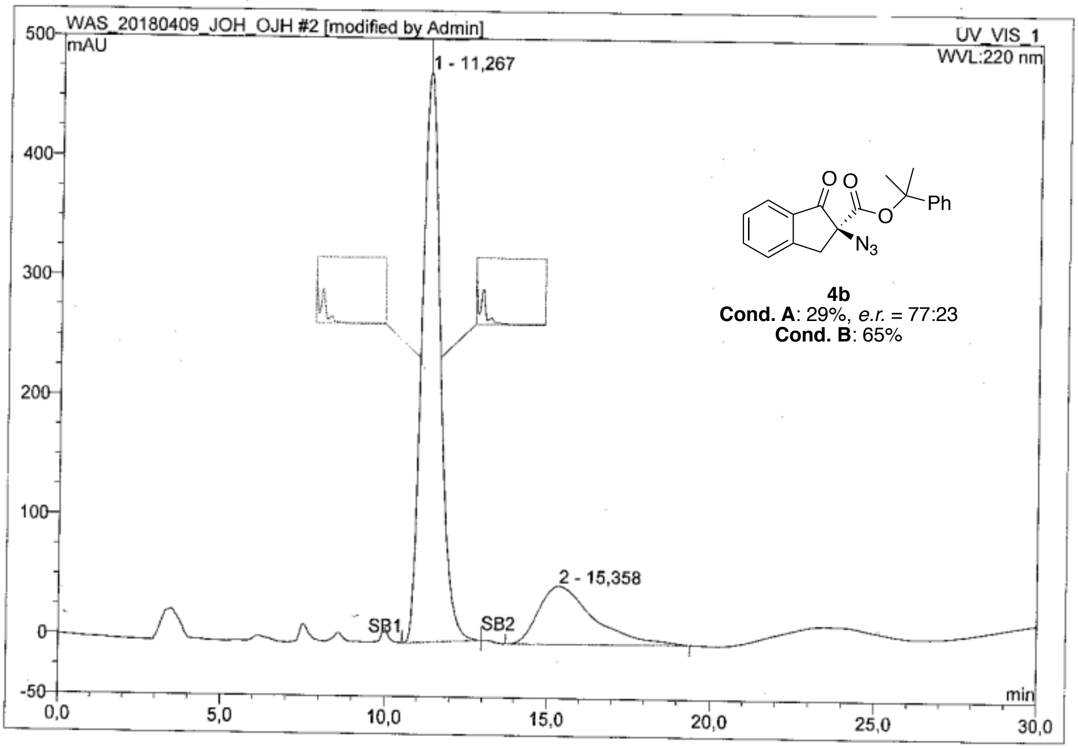

No.	Ret.Time min	Peak Name	Height mAU	Area mAU*min	Rel.Area %	Amount	Type
1	11,27	n.a.	475,574	312,561	76,52	n.a.	BMB
2	15,36	n.a.	48,497	95,892	23,48	n.a.	BMB
**Total:**			524,071	408,453	100,00	0,000	

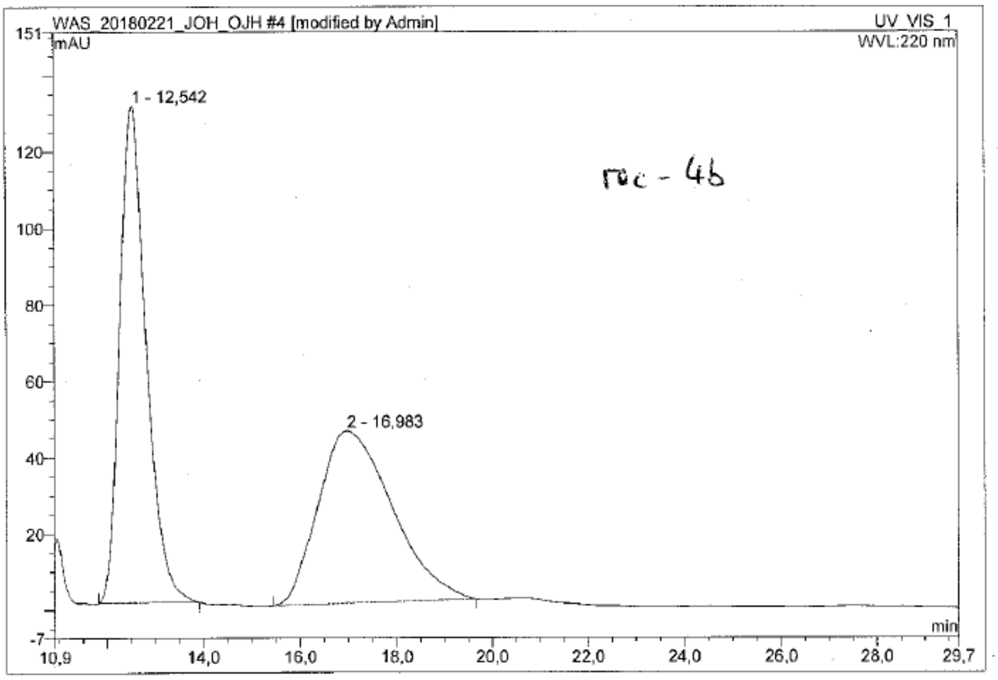

No.	Ret.Time min	Peak Name	Height mAU	Area mAU*min	Rel.Area %	Amount	Type
1	12,54	n.a.	130,068	83,164	50,78	n.a.	BMB*
2	16,98	n.a.	45,190	80,616	49,22	n.a.	BMB*
**Total:**			175,258	163,780	100,00	0,000	

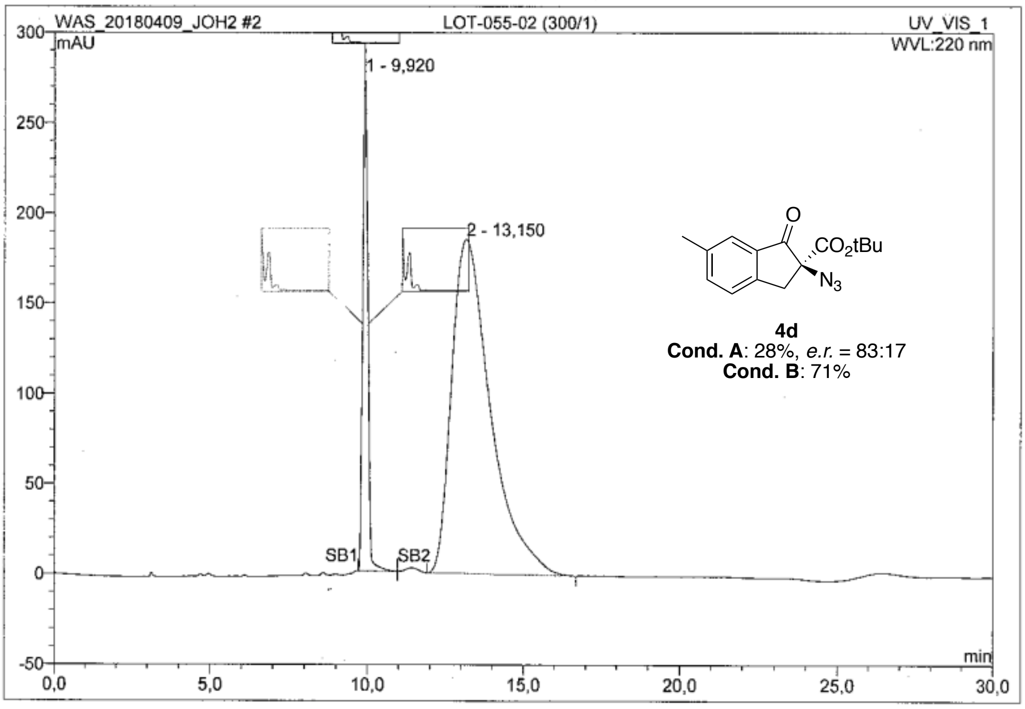

No.	Ret.Time min	Peak Name	Height mAU	Area mAU*min	Rel.Area %	Amount	Type
1	9,92	n.a.	274,944	53,558	16,61	n.a.	BMB
2	13,15	n.a.	185,246	268,957	83,39	n.a.	BMB
Total:			460,189	322,515	100,00	0,000	

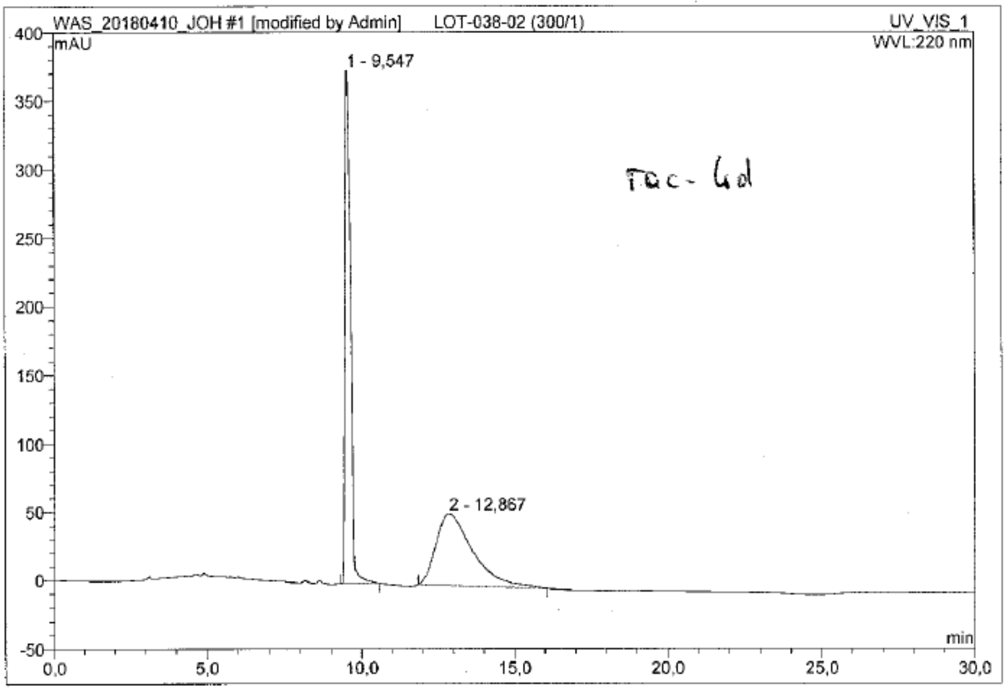

No.	Ret.Time min	Peak Name	Height mAU	Area mAU*min	Rel.Area %	Amount	Type
1	9,55	n.a.	374,952	77,716	51,35	n.a.	BMB*
2	12,87	n.a.	52,645	73,638	48,65	n.a.	BMB*
Total:			427,596	151,353	100,00	0,000	

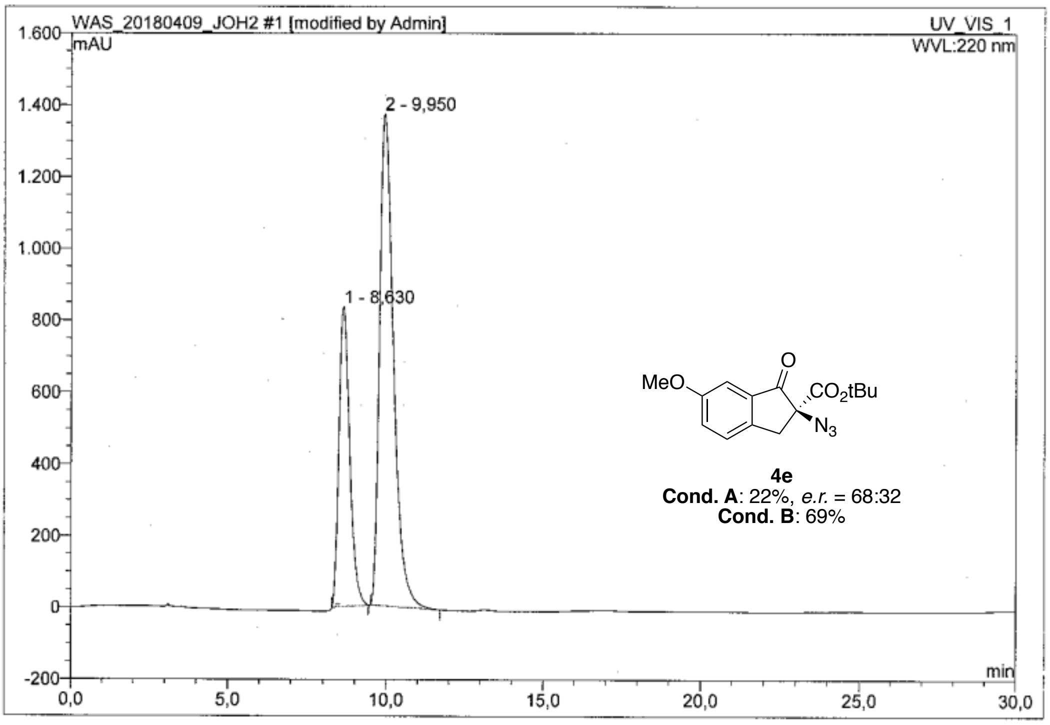

No.	Ret.Time min	Peak Name	Height mAU	Area mAU*min	Rel.Area %	Amount	Type
1	8,63	n.a.	834,845	329,267	32,02	n.a.	BMB*
2	9,95	n.a.	1370,991	699,115	67,98	n.a.	BMB*
Total:			2205,836	1028,382	100,00	0,000	

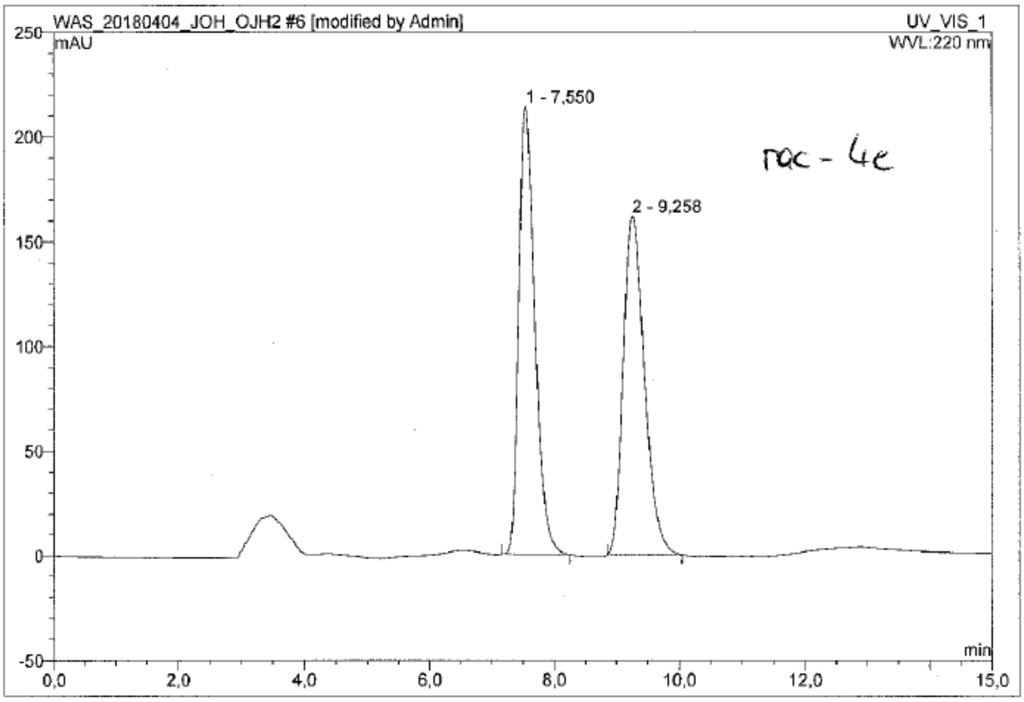

No.	Ret.Time min	Peak Name	Height mAU	Area mAU*min	Rel.Area %	Amount	Type
1	7,55	n.a.	213,831	63,932	50,21	n.a.	BMB*
2	9,26	n.a.	161,695	63,408	49,79	n.a.	BMB*
Total:			375,526	127,340	100,00	0,000	

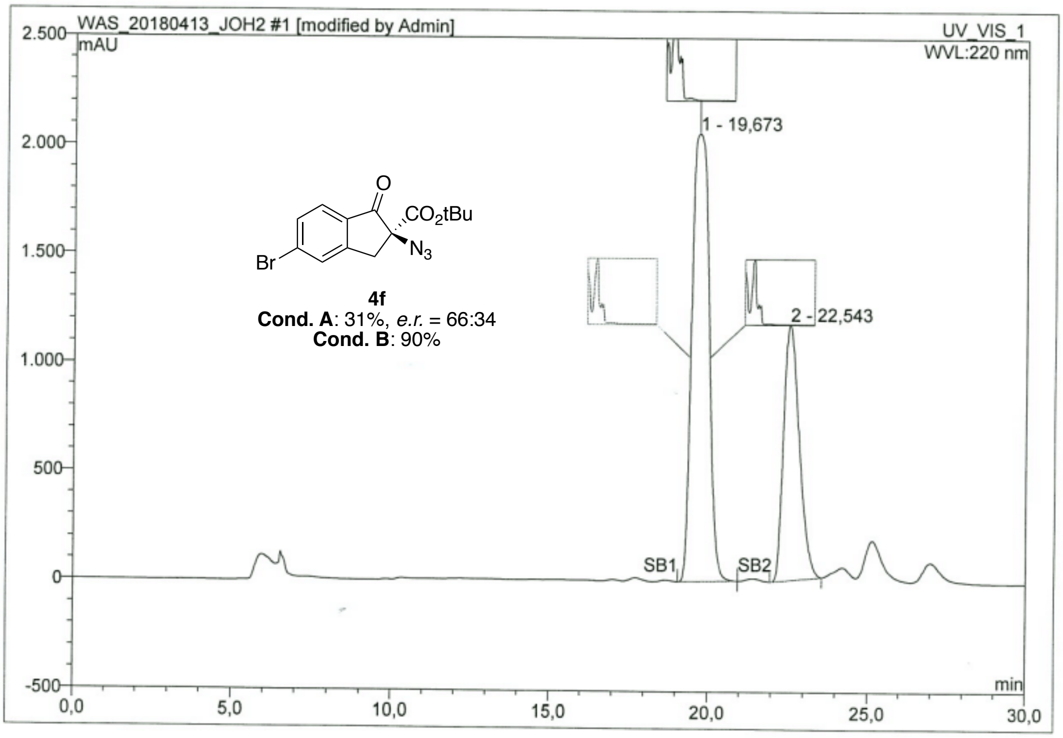

No.	Ret.Time min	Peak Name	Height mAU	Area mAU*min	Rel.Area %	Amount	Type
1	19,67	n.a.	2059,200	1285,551	65,73	n.a.	BMB*
2	22,54	n.a.	1174,848	670,333	34,27	n.a.	BMB*
Total:			3234,049	1955,884	100,00	0,000	

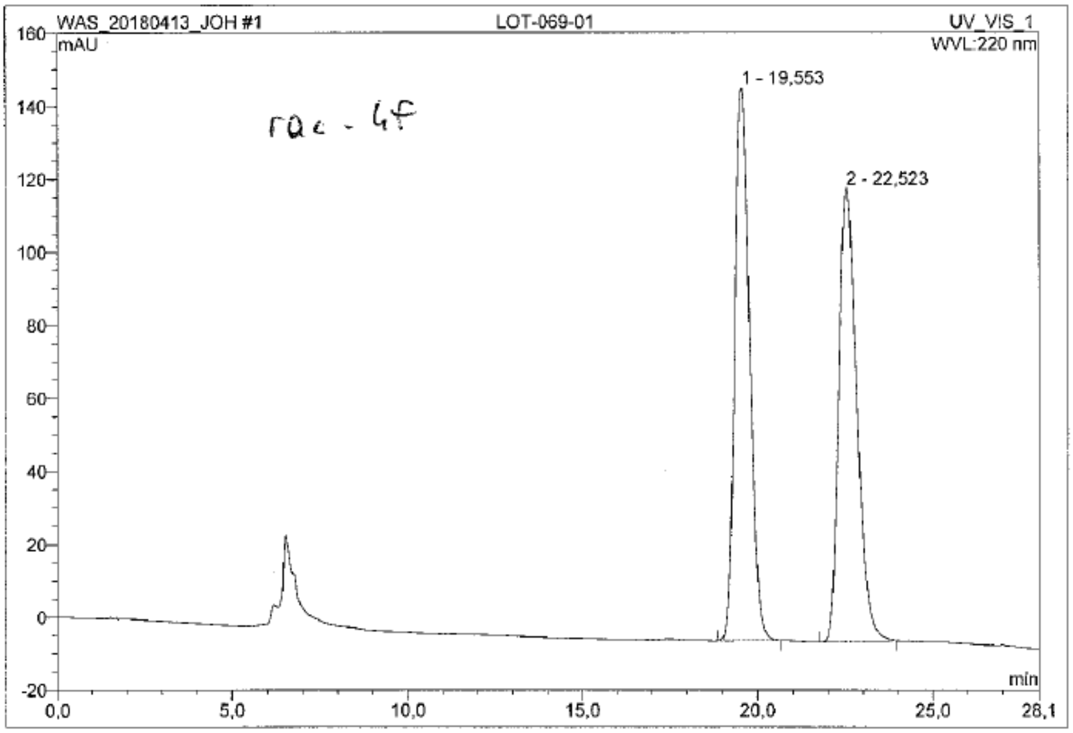

No.	Ret.Time min	Peak Name	Height mAU	Area mAU*min	Rel.Area %	Amount	Type
1	19,55	n.a.	151,468	74,597	50,35	n.a.	BMB
2	22,52	n.a.	124,335	73,558	49,65	n.a.	BMB
Total:			275,802	148,154	100,00	0,000	

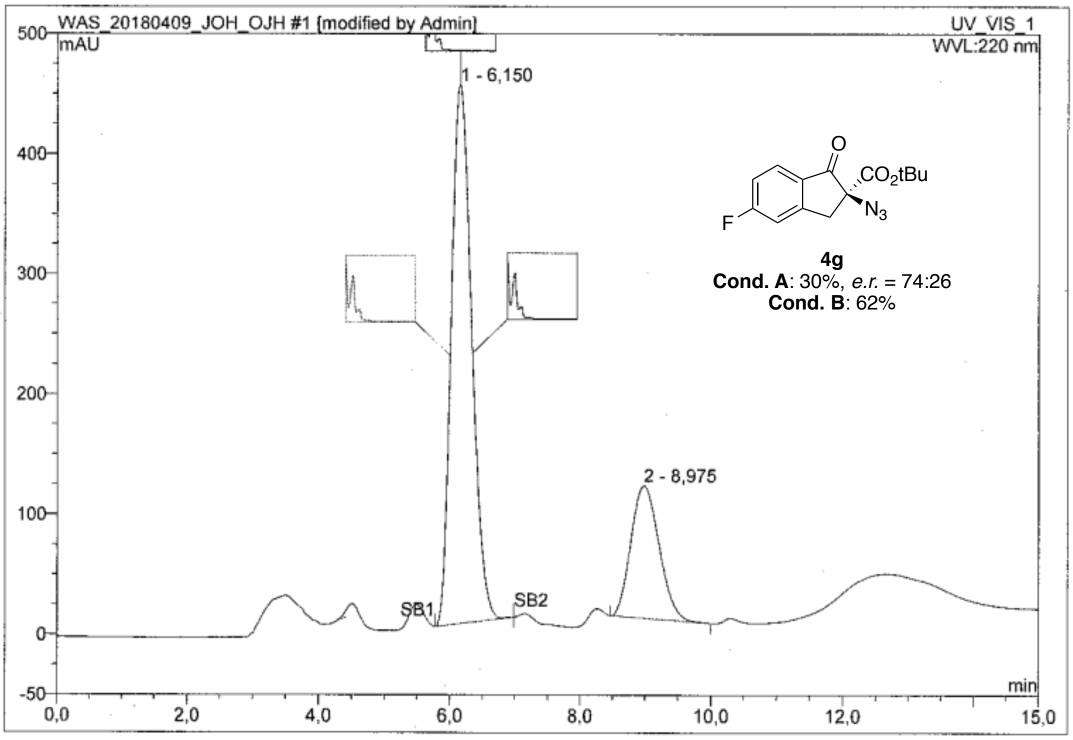

No.	Ret.Time min	Peak Name	Height mAU	Area mAU*min	Rel.Area %	Amount	Type
1	6,15	n.a.	448,114	162,243	74,48	n.a.	BMB
2	8,98	n.a.	110,395	55,577	25,52	n.a.	BMB
Total:			558,509	217,821	100,00	0,000	

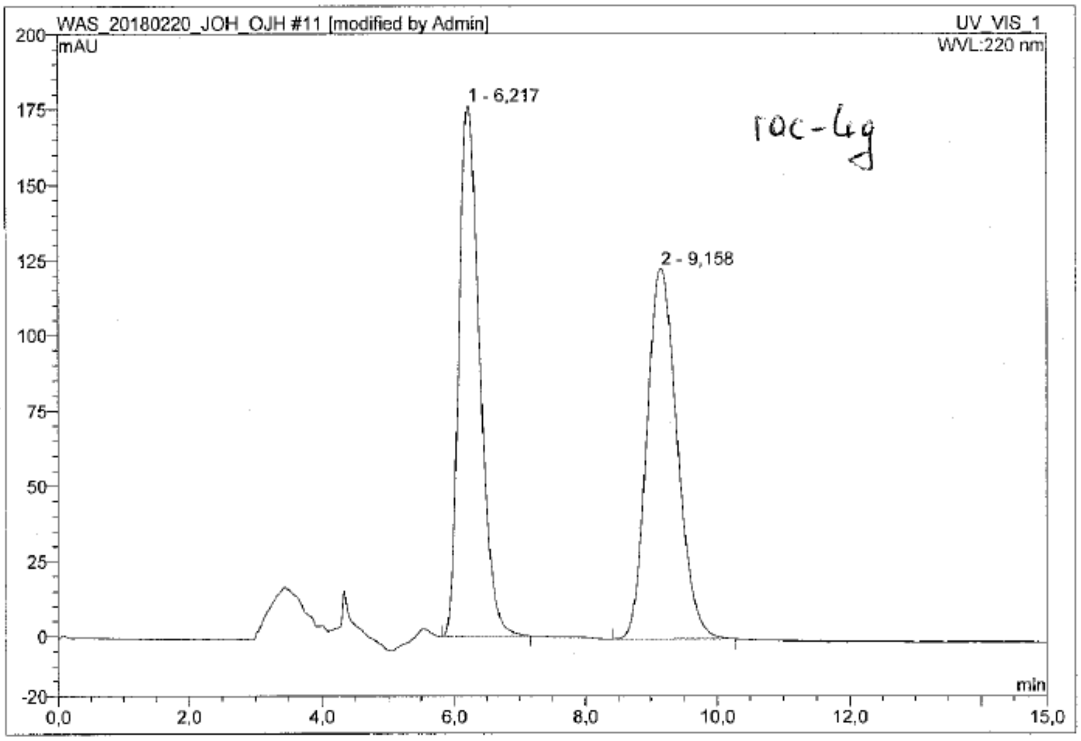

No.	Ret.Time min	Peak Name	Height mAU	Area mAU*min	Rel.Area %	Amount	Type
1	6,22	n.a.	176,313	65,272	49,83	n.a.	BMB*
2	9,16	n.a.	123,402	65,723	50,17	n.a.	BMB*
Total:			299,715	130,996	100,00	0,000	
